# Printing and Coating Techniques for Scalable Organic Photovoltaic Fabrication

**DOI:** 10.3390/ma17112511

**Published:** 2024-05-23

**Authors:** Bradley P. Kirk, Jonas M. Bjuggren, Gunther G. Andersson, Paul Dastoor, Mats R. Andersson

**Affiliations:** 1 Flinders Institute for Nanoscale Science and Technology, College of Science and Engineering, Flinders University, Sturt Road, Bedford Park, Adelaide, SA 5042, Australia; 2 Centre for Organic Electronics, University of Newcastle, University Drive, Callaghan, NSW 2308, Australia

**Keywords:** organic photovoltaics, printing and coating, scalability

## Abstract

Within recent years, there has been an increased interest towards organic photovoltaics (OPVs), especially with their significant device performance reaching beyond 19% since 2022. With these advances in the device performance of laboratory-scaled OPVs, there has also been more attention directed towards using printing and coating methods that are compatible with large-scale fabrication. Though large-area (>100 cm^2^) OPVs have reached an efficiency of 15%, this is still behind that of laboratory-scale OPVs. There also needs to be more focus on determining strategies for improving the lifetime of OPVs that are suitable for scalable manufacturing, as well as methods for reducing material and manufacturing costs. In this paper, we compare several printing and coating methods that are employed to fabricate OPVs, with the main focus towards the deposition of the active layer. This includes a comparison of performances at laboratory (<1 cm^2^), small (1–10 cm^2^), medium (10–100 cm^2^), and large (>100 cm^2^) active area fabrications, encompassing devices that use scalable printing and coating methods for only the active layer, as well as “fully printed/coated” devices. The article also compares the research focus of each of the printing and coating techniques and predicts the general direction that scalable and large-scale OPVs will head towards.

## 1. Introduction

With the drive towards environmentally friendly electricity production, there has been increased interest towards the research and development of renewable energy sources [1,2]. The goal for future energy sources is to provide electrical power that minimises environmental impact [3,4]. Renewable energy is defined as an energy generated from sources that can be replenished on a human timescale [5]. One such promising energy source, photovoltaic (PV) cells, utilises solar energy by directly converting the energy from sunlight into electricity. For nations with a high average sun intensity such as Australia, converting solar energy into power with PV devices can allow for cheap and renewable energy. From 2012 to 2022, there was a global growth of 1140% in solar energy production, whereas for wind generation there was an increase of 277% [6].

As solar has become a greater proportion of green energy generation, there has been interest directed towards the development of a third generation of photovoltaics, devices which are able to be printed on lightweight substrates, allowing for a reduction in manufacturing costs. One such type of third-generation photovoltaics, known as organic photovoltaics (OPVs), utilises conjugated polymers and/or molecules to generate direct current electricity. To achieve this, light is absorbed by the active layer materials, resulting in the generation of excitons, before these are split into free charges (i.e., electrons and holes). Due to their relatively low bandgap, lower-energy photons are able to be absorbed by the OPV devices, enabling the effective collection of light photons across the visible spectrum. In addition, due to their improved efficiency at low irradiance, OPVs also function effectively in indirect sunlight [7] and indoor conditions from artificial lighting [8,9,10].

Over the past 10 years, there has been significant development in the field of OPVs, from between an 8 and 9% performance power conversion efficiency (PCE) in 2012 [11,12] to an increase beyond a 19% performance conversion efficiency since 2022 [13,14,15]. These advances can be attributed to extensive work on the development of novel conjugated polymers and small-molecule acceptors, as well as the ability to fine-tune the active layer morphology. Yet, these improvements have primarily been focused on single-layered laboratory-scaled devices, which utilise a non-vacuum deposition technique known as spin-coating. Though the technique is relatively cheap, easy to use, and can produce highly reproducible films, it is not suitable for large area fabrication due to its non-uniform film formation when prepared on large substrates, resulting in a reduction in device performance. As such, there have been several printing and coating techniques that have been explored for the scalable and large-scale fabrication of said devices. These include:

- Rod coating
- Blade coating
- Slot-die coating
- Spray coating
- Pad printing
- Ink-jet printing
- Gravure printing
- Flexographic printing
- Screen printing

These non-vacuum-based printing and coating methods are less energy intensive compared to vacuum-based techniques, while simultaneously allowing for facile manufacturing, thus reducing production cost and energy payback time. With these benefits in mind, researchers and industry have aimed to transition OPV technology from laboratory-based fabrication to large-scale manufacturing. To achieve this, there has been a focus towards determining coating methods and conditions that can be applied to a roll-to-roll (R2R) printing/coating system.

Though there have been significant strides in device performance, other areas such as material cost and complexity, scalability, and stability have not seen as much focus. Approaches to improving stability and cost have mainly focused on laboratory-scale devices, and there is some level of uncertainty associated with their scalability to large-area OPVs that have been fabricated using alternative coating methods. Despite the main focus on laboratory-scale OPVs, recent years have seen significant improvements in upscaling. Recently, devices with an active area above 100 cm^2^ now reach an over 15% PCE [16].

The focus of this paper is to explore and compare several non-vacuum printing and coating processes that have been demonstrated in OPV fabrication, including the materials and layers that have been coated, the performances achieved, and the active area that has been attempted. To simplify the comparison, active areas are categorised as laboratory (<1 cm^2^), small (1–10 cm^2^), medium (10–100 cm^2^), and large (>100 cm^2^) scale. The review also focuses on the progression of printing and coating techniques over the decade, their popularity in the field of OPVs, and the challenges that they need to overcome. To allow for concise comparisons between printing and coating conditions, devices will be categorised by the coating technique utilised for active layer deposition. It is worth highlighting that combinations of techniques have been utilised for the coating deposition of transport and interface layers, as well as electrodes. Lastly, though the cost of OPV fabrication is not the focus of this paper, we as authors are aware of its significance in the cost of upscaling and commercialisation. 

## 2. Background

**Figure 1:**
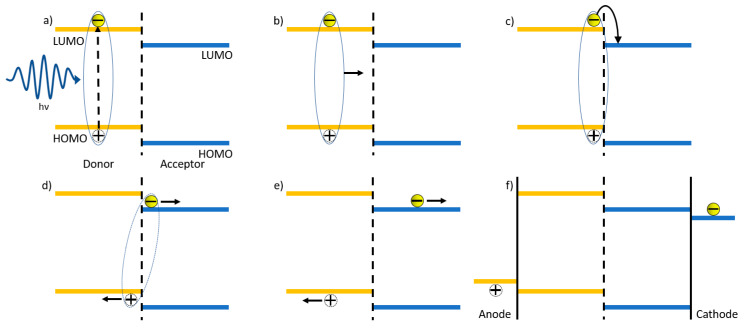
Schematic of photocurrent generation in OPVs: (**a**) absorption of a photon to form an exciton, (**b**) exciton diffusion towards the acceptor-donor interface, (**c**) exciton dissociation, (**d**) charge carrier separation, (**e**) charge carrier movement through the active materials, and (**f**) the collection of charge at their respective electrodes. The grey ellipse indicates bound electron and hole pair.

The electrical properties of conjugated polymers arise from the alternating single–double bond structuring of the sp^2^ hybridized carbon atoms present in the backbone of the polymer [17]. Due to this structuring, conjugated polymers exhibit strong light absorption, allowing for the excitation of electrons from the ground state to an excited state, with the overlap of the highest occupied molecular orbitals (HOMOs) and lowest unoccupied molecular orbitals (LUMOs) resulting in the formation of valence and conduction bands, respectively. The HOMO-LUMO energy difference is defined as the bandgap and corresponds to the lowest energy required to excite an electron from the valence to the conduction band. This bandgap can easily be altered through the tuning of the polymer structure [18,19]. To enable efficient exciton dissociation, donor polymers are typically paired with accepter materials that facilitate both charge separation and electron transport (seen in Figure 1).

Conjugated acceptor materials that have been investigated include, but are not limited to, neat C_70_ [20], [6,6]-phenyl-C61-butyric acid methyl ester (PC_61_BM) [21], [6,6]-phenyl-C71-butyric acid methyl ester (PC_71_BM) [22], 2,2′-[[6,6,12,12-Tetrakis(4-hexylphenyl)-6,12-dihydrodithieno [2,3-d:2′,3′-d′]-s-indaceno [1,2-b:5,6-b′]dithiophene-2,8-diyl]bis[methylidyne(3-oxo-1H-indene-2,1(3H)-diylidene)]]bis[propanedinitrile] (ITIC) [23,24,25], poly{[N,N′-bis(2-octyldodecyl)naphthalene-1,4,5,8-bis(dicarboximide)-2,6-diyl]-alt-5,5′-(2,2′-bithiophene)} (N2200) [26,27], and 2,2′-[[12,13-Bis(2-ethylhexyl)-12,13-dihydro-3,9-diundecylbisthieno [2″,3″:4′,5′]thieno [2′,3′:4,5]pyrrolo [3,2-e:2′,3′-g][2,1,3]benzothiadiazole-2,10-diyl]bis[methylidyne(5,6-difluoro-3-oxo-1H-indene-2,1(3H)-diylidene)]]bis[propanedinitrile] (Y6) [28,29,30].

Over the decades, there has been an effort towards improving the design of the physical structuring of devices, specifically the design of the active layer. The first generation of OPVs used a single layer structure in between two metal electrodes. This was found to yield extremely low efficiencies, as the charge separation was insufficient for these devices [31]. Since then, there have been studies focusing on single layer materials that contain both donor and acceptor segments within their molecular structure [32,33,34,35]. To date, the highest efficiency achieved with this type of single layer material is just above 13% [35].

The second generation of OPVs employed a bilayer for their devices. First published in 1980, the bilayer planar heterojunction consists of donor and acceptor materials [31]. With the use of donor and acceptor materials, it allows for the separation of the electron–hole pair at the domain interface, resulting in a reduction in pair recombination, allowing for an increased performance and higher output of energy. Though an improvement over single layered devices was evident, their efficiencies were still relatively low at 1%. This low efficiency was associated with the exciton diffusion length, which limits the number of excitons reaching the donor–acceptor interface and, hence, the charge carrier formation, as well as efficiency [36]. Despite these disadvantages, an impressive performance of over 7% has been achieved with a “bilayer” device [10].

**Figure 2:**
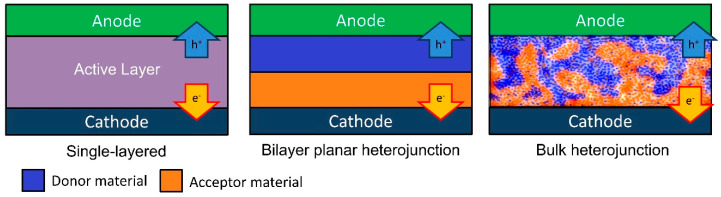
A basic model of types of junction structure: single, bilayer planar, and bulk heterojunction solar cell.

The third generation of OPVs had bulk heterojunction (BHJ) structuring for the active layer, focusing on the intermixing of acceptor–donor domains. The BHJ layer involves the polymer donor and acceptor (fullerene, non-fullerene, and polymer) materials being blended into a single film. First developed in 1995, it had been shown that the BHJ is a superior design compared to a bilayer [37], where the acceptor and donor materials appear in separate layers. The intermixing of materials creates an increased interfacial surface area (Figure 2) where the separation of electron–hole pairs can occur.

For the BHJ to work efficiently as an active layer, there must be both good mixing and good miscibility between the polymer and fullerene material [38].

Nowadays, there is a shift towards pseudo-bilayers [10,39] and sequentially deposited [15,40,41,42] active layers. The advantage of this approach is that it enables better control over the interfacial area between the donor and acceptor, depending on the solvent used in the top active material ink and film processing. Sequential deposition has, so far, achieved a record PCE exceeding 19% [13,15].

There are two main types of device architecture: conventional and inverted device structures, with both arrangements having photons incident from the bottom (i.e., through the substrate) but differing in polarity. The structure order is as follows:

Conventional: substrate **→** anode **→** hole transport layer (HTL) **→** active layer **→** electron transport layer (ETL) **→** cathode

Inverted: substrate **→** cathode **→** electron transport layer (ETL) **→** active layer **→** hole transport layer (HTL) **→** anode

**Figure 3:**
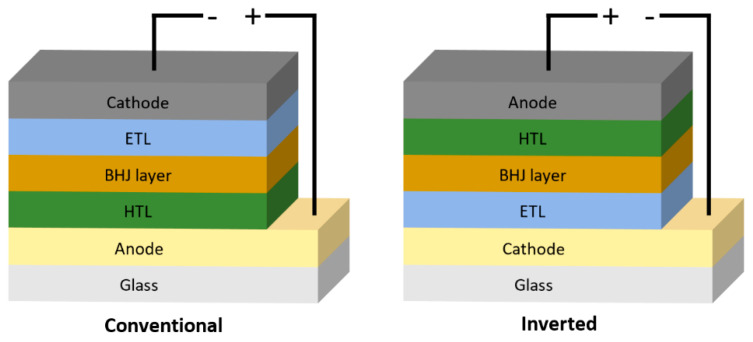
Schematic of conventional (**left**) and inverted (**right**) devices.

These two device structures can be seen in the schematic below (seen in Figure 3). It is noted that the transport layers can also act as a buffer or blocking layer.

In conventional OPV devices, indium tin oxide (ITO) is used as the anode, allowing for the collection of holes and for light to pass through to reach the active layer. Poly(3,4-ethylenedioxythiophene):poly(4-styrenesulfonate) (PEDOT:PSS) is commonly applied between the anode and the active layer [43,44,45]. It serves to modify the work function of the anode, act as a hole-transport layer (HTL), and smooths the anode topography [46,47,48]. It can also act as a barrier between the anode and active layer to limit the diffusion of ITO into the BHJ [49], which would otherwise reduce the device efficiency [49]. Post-annealing of the PEDOT:PSS allows for the removal of moisture from the film, improving the conductivity and, thus, the performance of devices [50].

Layered over the active layer, either lithium fluoride (LiF) [51] or calcium (Ca) [52] are commonly used as a cathode buffer layer to improve devices. These materials are generally processed via thermal evaporation, thus resulting in an increase in fabrication cost [53]. There are, however, materials that can be deposited via non-vacuum processes, including but not limited to zinc oxide (ZnO) [54,55], titanium oxide (TiO_X_) [56,57], tin oxide (SnO_2_) [58,59], polyethyleneimine (PEI) [60], and polyethyleneimine ethoxylated (PEIE) [61]. For printed/coated devices, zinc oxide nanoparticles (ZnO NPs) can be used as a popular alternative as they are solution processable, allowing for ease of deposition as an ETL without requiring high-temperature post-annealing of the film [62]. Lastly, aluminium (Al) is commonly positioned on top to allow for the collection of electrons [44].

In inverted OPV devices, the ITO is used as the cathode, allowing for the collection of electrons. ZnO is commonly used as the cathode buffer layer/electron transport layer, allowing for the lower work function of the ITO, thus improving the anode alignment with the LUMO energy level of the acceptor material [63,64,65]. Above the active layer, molybdenum oxide (MoO_X_) is used to increase the work function of the anode, as well as act as a HTL [66,67].

Though this can improve the efficiency of laboratory-scale devices, it is not desired for large-scale roll-to-roll fabrication, due to having to involve sputtering techniques for MoO_X_ deposition [53]. To resolve this, there has been work conducted towards solution-processable MoO_X_ [68,69,70], which has been demonstrated by depositing via blade coating [70]. PEDOT:PSS has been used as a popular alternative as it can be coated/printed over the active layer [63], however, there are issues with its stability.

In the literature, other materials have also been demonstrated as effective alternatives, including but not limited to vanadium oxide (V_2_O_5_) [71,72], graphene oxide [73,74], and nickel oxide (NiO_X_) [75,76]. Lastly, either aluminium (Al) or silver (Ag) are used as the anode for inverted devices [77]. Inverted devices are normally more stable than conventional OPVs [78,79,80].

## 3. Coating and Printing Techniques for OPV

Another area of importance for the commercialisation of OPV is related to the development of scalable and upscaled coating and printing techniques. Since 2008, several scalable coating and printing techniques have been investigated, focusing on either reducing the performance gap between lab-based cells, large-area cells, and even modules, while other groups look towards reducing the complexity and environmental impact of OPV fabrication.

### 3.1. Vacuum and Non-Vacuum Deposition

When discussing coating and printing techniques, they are divided into two areas: vacuum-based and non-vacuum-based techniques. As the name suggests, vacuum processes involve techniques that require a high vacuum to perform material deposition. Depending on the material, either thermal evaporation or sputter coating are employed. Materials that are commonly deposited via vacuum deposition include metal oxides (MoO_X_ [59,81,82], LiF [83,84]), and metal electrodes (Ag [85,86], Al [87,88]). These techniques are useful for the deposition of thin film materials (<50 nm) without exposing the layer to oxygen/moisture, which would otherwise result in device degradation. Some studies have focused on depositing these materials using non-vacuum processes, with Ag paste printing being the most used in these attempts [82,89,90]. As for metal oxides, there have been attempts to transfer previously vacuum-processed materials to solution-processed materials, such a MoO_X_ [91], and alternative materials, such as SnO_2_ [59], V_2_O_5_ [92,93] and PEDOT:PSS [90,94]. There has also been interest in the development of vacuum-processed active layers, a method that deposits photoactive organic molecules to form the active layer of the OPV [95,96,97,98], yet with limited success compared to either solution-processed counterparts.

The major issue associated with vacuum processing is its capital and running costs, requiring complex machinery, which leads to high initial costs and high electricity costs due to the amount of energy required to form the vacuum and deposit the materials. In saying that, the high precision and control of deposition thickness cannot be understated when compared with some non-vacuum methods.

Non-vacuum-processed (also known as solution-processed) techniques are generally less complex and more cost-effective compared to vacuum-processed techniques. To control the thickness of the layers, the concentration, as well as the printing/coating conditions, can be optimised. Depending on the method, however, some issues with non-vacuum deposition can include the layers being exposed to oxygen/moisture, a lack of precise control of the layer thickness, and a lack of pattern control. These issues are heavily dependent on printing/coating techniques (as discussed in Section 3.4), with post-processing being able to resolve some issues.

In general, the majority of laboratory-, small-, medium-, and large-scale OPV fabrication incorporates a combination of non-vacuum techniques to deposited layers, depending on the layer composition and thickness control. It is worth noting that the scalability of vacuum-based processes that are large-scale roll-to-roll compatible has been demonstrated [44,98,99].

### 3.2. OPV Fabrication Scalability via Alternative Printing and Coating Techniques

Most publications compare the scalability of fabrication methods based on the idea that printing/coating techniques can be performed via roll-to-roll fabrication. However, some methods, such as sheet-to-sheet, sheets-on-shuttle, and and roll-to-sheet, can still yield large-scale OPV fabrication, rather than requiring a continuous roll fabrication [100]. Overall, roll-to-roll is ideal for the fabrication of flexible OPV devices, whereas flat-bed is more ideal for rigid OPV devices (such as for windows).

Another important aspect is associated with the scalability of the active area, and the definition of the cell vs. modules. Firstly, an active area is defined as the area of the device that is being exposed to light, resulting in exciton generation. It is common to use laboratory active areas (~0.1 cm^2^) to yield a higher performance, as this minimises the impact of film defects that would otherwise result in a PCE reduction. For demonstrating scalability, larger active areas ranging from laboratory, small, medium, and large areas have been employed.

Secondly, a cell is defined by a single OPV device that, when under operating conditions, is not connected to any other PV device (neither in series nor parallel). These cells can range from having an active area of 0.4 cm^2^ [101,102] to 18 cm^2^ [103] and are commonly used individually to maximise device performance. Lastly, when wanting to demonstrate the efficiency of large-area OPVs, it is common practice to fabricate modules, like a multi-cell device which can be connected either in parallel or series (depending on the groups conducting the research). For OPVs, the active area of these modules found in the literature can range from 2 cm^2^ [104] to 360 cm^2^ [90].

Lastly, it is important to address the complexity of increasing the active layer area, and why there appears to be a performance gap between laboratory and large-area devices. Issues such as the low-sheet resistance of materials (especially the electrodes) [105,106,107] and non-uniform film quality and defects [108,109] can lead to a reduction in the charge generation and transport within the device [110,111,112], leading to a reduction in the overall performance. As such, not only should the focus be on the development of ideal materials for OPV devices, but also the development of printing and coating techniques to allow for uniform, defect-free, and consistent film quality.

### 3.3. Roll-to-Roll Fabrication

Whenever discussing the upscaling of OPV fabrication, it is important to mention roll-to-roll processing. Roll-to-roll processing usually involves a transparent, flexible substrate that is fed from one roll to another, with a series of coating processes and pre/post processes interacting with said substrate. For roll-to-roll, it can be categorised into two parts, modular and inline.

**Figure 4:**
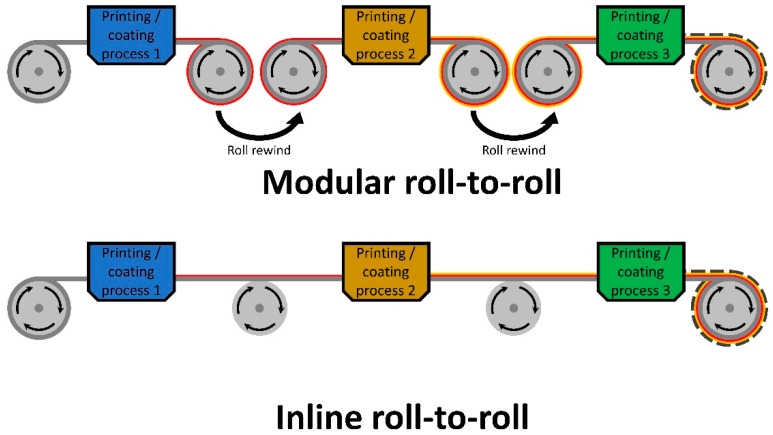
Basic schematics of the type of roll-to-roll fabrication procedures.

For modular, a single printing/coating process is conducted during the rewinding of the substrate, resulting in a single thin layer being deposited prior to the rewinding of the roll (seen in Figure 4).

The advantage of this method is that it is incorporated in laboratory-scale OPV fabrication, while only requiring a medium amount of lab space. This method also allows for optimised coating conditions to be implemented during each layer deposition. The only noted issue with the technique is that the fabrication time is greatly increased due to the time taken to set up for the next deposition process. A few groups have employed this method of roll-to-roll fabrication [90,113], as it is more suitable for laboratory conditions.

As for inline roll-to-roll fabrication, multiple coating processes are conducted on a single wind of the substrate (seen in Figure 4), allowing for a reduction in the overall fabrication time, thus making this technique favourable for commercial conditions. One setback is that the winding speed is required to be the same for all coating processes, which may be an issue if the optimal coating is not the same for all material depositions. This technique has been performed by a few research groups during the past decade [114,115].

### 3.4. Types of Printing and Coating Methods

In the past 15 years, there has been a significant increase in efforts directed towards the development and implementation of printing and coating techniques that not only allow for the fabrication of high-performance OPVs, but also devices with large active areas, minimal film defects, and reduced manufacturing costs. As such, there are several methods that have been shown to be key in solving this goal, each with their own benefits and challenges. In this section, several types of solution-processed printing and coating techniques will be discussed, highlighting past and current developments and their popularity in the OPV field. When discussing the pattern control of printing and coating conditions, coating methods are only able to adjust the film thickness (zero-dimensional) or both the strip width and thickness (one-dimensional), while printing can form patterns along the XY-plane, as well as the thickness (two-dimensional). The scale of devices is defined by the active area that is used for device testing, with areas being categorised into laboratory (<1 cm^2^), small (1–10 cm^2^), medium (10–100 cm^2^), and large (>100 cm^2^) scale.

#### 3.4.1. Spin Coating

**Figure 5:**
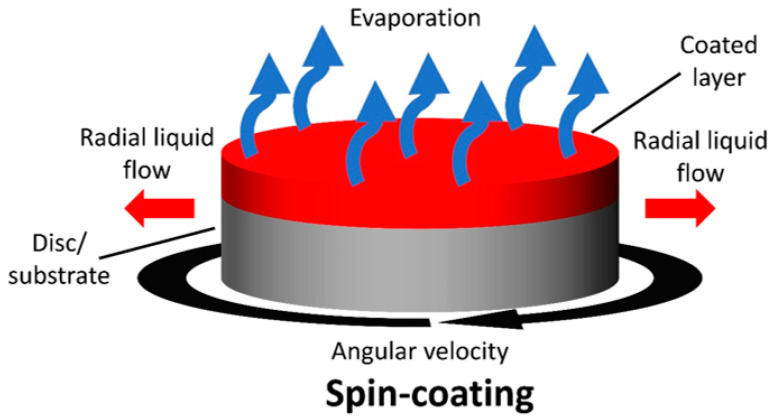
Basic schematic of spin coating. Adapted from Krebs, 2009 [111].

Spin coating is a zero-dimensional, non-roll-to-roll compatible coating method that requires spinning a substrate at high speeds (800 to 5000 rpm) to allow for the formation of thin films (seen in Figure 5).

The ink can be placed on the spinning substate either before or during the spinning process. The thickness of the film can be controlled by adjusting the spin speed, acceleration, coating time, and solution concentration. Due to the simplicity of the method for producing laboratory-scale devices, it is one of the most common methods employed in labs to fabricate OPVs. This method has also yielded the highest-efficiency OPVs when compared to other coating methods [13,116,117,118]. The major issue with this method is its limited scalability [114,119]. However, due to the higher power conversion efficiencies achieved by this process, it is typically used as a benchmark comparison for other printing/coating methods.

Owing to its simplicity as a coating method, as well as the ability to fabricate high-performing devices consistently, a wide range of materials have been coated using this technique. This includes several active layer blend combinations (i.e., P3HT:PCBM [120,121,122], PBDB-T:ITIC [123,124,125], PM6:Y6 [29,126,127]), electron transport layers (i.e., ZnO [128,129], AZO [130,131,132]), and hole transport layers (i.e., PEDOT:PSS [133,134], V_2_O_5_ [72]). To date, the highest efficiency for a single-junction OPV that has been achieved is 19.3 ± 0.1%, with the device using PM6:D18:L8-BO as the photoactive material [13].

Overall, spin coating is one of the most common coating methods used in OPV research due to its reproducibility and ability to achieve high device performances. Despite their lack of scalability, spin-coated devices are still useful for comparison with scalable and large-scale OPVs, as well as in the research of novel materials, processing, and ink preparation.

#### 3.4.2. Rod Coating

**Figure 6:**
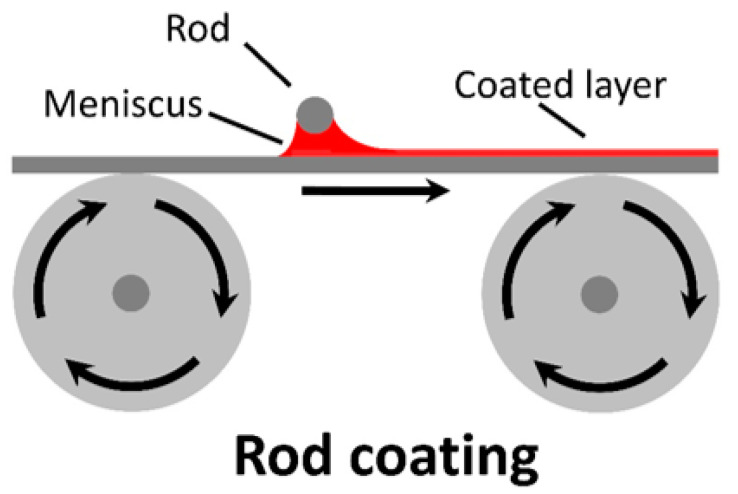
Basic schematic of rod coating. Adapted from Søndergaard et al., 2012 [135].

Rod coating is a zero-dimensional, contactless coating method that works by forming a meniscus between the rod and substrate from the coating ink (seen in Figure 6).

Coating occurs as either the rod or substrate move, resulting in ink removal from the meniscus. From an extensive exploration of the literature, at least one attempt using rod coating for active layer deposition was found, with P3HT:PCBM being the active layer blend, yielding an efficiency of 1.9 ± 0.4%. All other applications of this technique have been used for Ag nanowires (AgNWs) [136], with this application aiming towards ITO-free substrates.

Although this technique is relatively simple to operate and automate, rod coating has seen little interest as a meniscus-based coating method, especially when compared to blade coating and slot-die coating (to be discussed later).

#### 3.4.3. Blade Coating/Knife-over-Edge Coating/Doctor Blading

**Figure 7:**
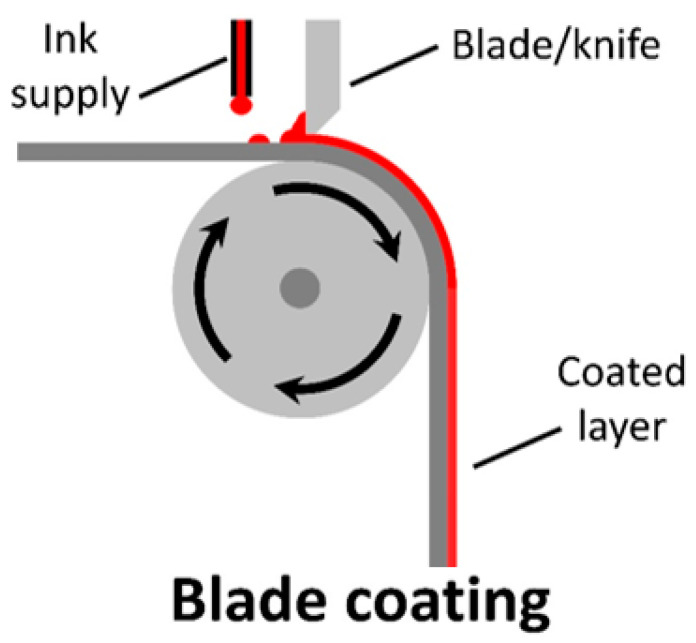
Basic schematic of blade coater. Adapted from Søndergaard et al., 2012 [135].

Blade coating is a zero-dimensional, contactless coating method that has minimal ink wastage and is roll-to-roll compatible. A build-up of ink on one side of the blade allows for the formation of a meniscus, where the thickness can be adjusted by controlling the substrate speed and blade–substrate gap (seen in Figure 7).

The final dry thickness of the blade-coated film (d) can be calculated from the following equation (Equation (1)):

(1) $$d=\frac{1}{2}\left(g\frac{c}{\rho }\right)$$

where g is the gap distance between the blade and substrate, c is the concentration of the solid material in the ink (g cm^−3^), and ρ is the material density in the final film (g cm^−3^).

Blade coating is one of the simplest methods for scalable and large-scale coating, only requiring a method for moving a substrate or a blade, and a method introducing ink between the blade and substrate. Due to its simplicity and ease of use, it has gained interest in large-scale fabrication, with many groups interested in the method.

The disadvantage of this method is that, besides the difficulty in controlling thickness, patterns are unable to be formed without employing an etching method (manual [84,137], chemical [64,138], or laser etching [81,139,140]). While there is research describing coated electrodes at the laboratory scale [141], the techniques’ limitations mean that, in general, blade coating is not ideal for electrode coating. Indeed, this technique is more suitable for flat-bed processing, resulting in the majority of research using rigid substrates [70,85,137,140,141,142,143,144,145,146,147,148,149], yet, it has been demonstrated that flat deposition on flexible substrates is possible [82,139,150].

Despite these limitations, there has been extensive research on reducing the performance gap between blade coating and spin coating [85,147,148,150], as well as device fabrication with higher active areas. To improve the coating quality, uniformity, and morphological control of the active layer, there are several examples of the implementation of in situ annealing [84,137,140,141,145,151,152,153], as well as the use of hot air upon the deposition of the film [84,137]. These methods have allowed for the fabrication of laboratory- [139,149], medium- [81,140,148,154,155], and large-scale [16,81,84,137,156] OPV devices. Though some blade-coated OPVs are prepared under nitrogen [143,145,146,147] and argon [85] conditions to allow for the best performance possible, a vast majority fabricate these devices under ambient conditions [139,140,141,143,144,149,150,153].

With the versatility that has been shown with blade coating, there are an array of active layer materials that have been coated with this method, including, but not limited to, P3HT:PCBM [101,102], PCDTBT:PC_71_BM [150,157], PBDB-T:ITIC [123], and PTB7-Th:PC_71_BM [70]. Other layers that have been demonstrated to be coated include the hole transport layer (i.e., PEDOT:PSS [141]), electron transport layer (i.e., ZnO NPs [141,144]), and electrodes (i.e., Ag [158], Ag nanotubes [141,144]). In terms of the number of layers printed or coated using alternative techniques, the majority of the literature focuses on only one layer, usually the active layer, with the remaining having either been spin-coated or deposited via vacuum-based processes [85,140,145,146,147,148,149,152]. Notwithstanding this observation, there have been successful demonstrations of multiple layers being blade coated, specifically, the coating of two [140,148,159] or three layers [82,137,139], and even all-printed devices [141].

With the increased interest in the development of large-area coating with blade coating, this has motivated the research field to use high-performance active materials at a variety of coating scales. This has allowed for performances to reach as high as 15.7% using “PV-X Plus”, with an active layer of 0.04 cm^2^ [140]. There has also been interest directed toward fabricating large-scale devices using blade coating, reaching an efficiency of 12.6% with an active area of 194.8 cm^2^, when using active materials of PM6:Y6:PC_61_BM [81]. This performance is much lower than the 16.2% reached when using spin coating for the same materials [160]. The highest performance for large-area devices was 15.08% with an active area of 204 cm^2^, using PM6:Y6-C12:PC_61_BM as an active layer material [16]. This significantly reduces the performance gap between the highest-performing laboratory-scale and large-scale OPVs.

These higher performances can be attributed to the use of specific solvents [85,101,146,147,149,151,161], solvent additives [85,123,145,153,161], ink preparation strategies [144,152,159], and ternary blend combinations [150]. There has also been interest in implementing active materials that are more suitable for blade coating, rather than those that perform better for spin casting, with more materials like PBDB-T:ITIC appearing to achieve a better performance when blade coated when compared their spin-coated counterparts [123,149]. Other work that has been conducted by blade-coated OPVs include methods for improving stability [123,139,140,148], the development of OPVs for indoor applications (a.k.a. light recycling) [139], ink preparation via halogen-free/environmentally friendly processing [149], and the coating of active layer nanoparticles [143].

Overall, blade coating has been demonstrated to be an effective method for coating active layers with an excellent film quality and uniformity control, while being effective at controlling active layer morphology. The progress of this coating technique has allowed for a significant performance reduction between laboratory- and large-scale devices.

#### 3.4.4. Slot-Die Coating

**Figure 8:**
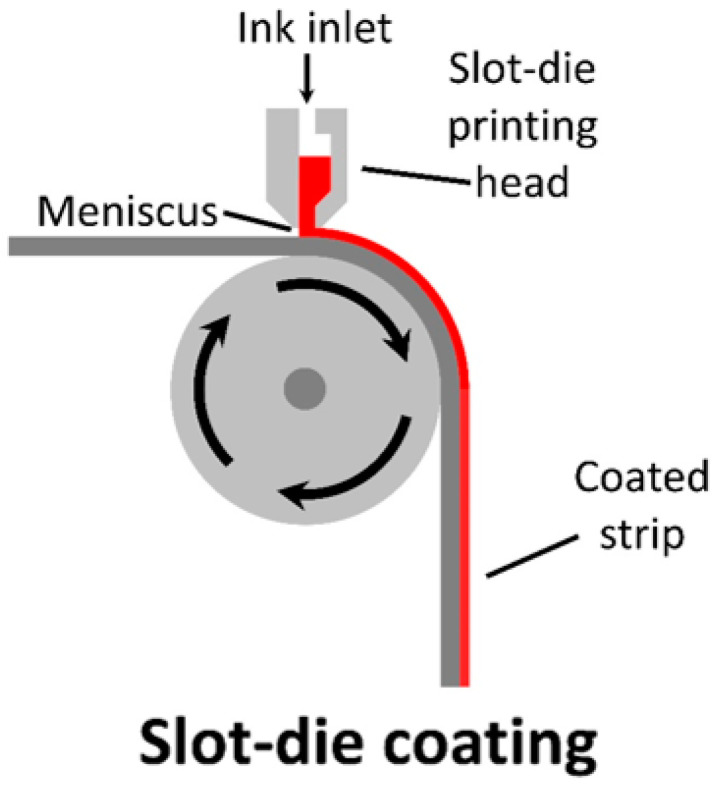
Basic schematic of slot-die coating. Adapted from Søndergaard et al., 2012 [135].

Slot-die coating is a one-dimensional, contactless coating method, which works by constantly supplying inking into a meniscus formed between the slot-die and substrate. Unlike blade coating (which coats the entire surface), the slot-die printing head can control the meniscus width, allowing for the coating of strips with a defined width. The slot-die printing head itself is relatively simple in design, with the ink being fed into a reservoir before flowing down to the meniscus (seen in Figure 8).

As the input of ink is controlled, there is a minimal wastage of ink. Unlike blade coating, where post-processing is required to form strips, the slot-die can control the meniscus width, allowing for the in situ formation of strips throughout the printing process.

For a given web, ink flow rate, coating width, and solid concentration, the final dry film thickness (d) can be estimated as follows (Equation (2)):

(2) $$d=\frac{f}{S\;w}\frac{c}{\rho }$$

where *d* is the thickness in cm, f is the flow rate in cm^3^ min^−1^, *S* is the web speed in cm min^−1^, *w* is the coated width in cm, *c* is the solid content in the ink in g cm^−3^ and, *ρ* is the density of the dried ink material in g cm^−3^.

Similar to blade coating, slot-die coating is a relatively simple method that is easily scalable, from rigid devices on a flatbed [59,63,104,113,142,162,163,164,165,166] to scalable devices coated from a mini-roll coater (MRC) [88,104,164,165,167,168,169] and even up to scalable and large-scale roll-to-roll fabrication [63,64,86,90,94,113,114,139,170,171,172,173]. With the variety of web-movement methods (flat-bed, MRC, and roll-to-roll), this allows an easier transition between scales, from the fabrication of laboratory-scale to medium-scale [65] and medium- to large-scale [167] OPV devices. This variability in scale has allowed for the fabrication and testing of OPV devices with increasing active areas, some reaching small [86,139,163,167,168,169,174], medium [87,90,94,104,114,165], and large scales [90,172].

It is worth noting that the vast majority of devices that have been investigated are fabricated over a flexible substrate [63,64,104,165,166,169,171,173,174], with a small percentage over a rigid substrate [59,63,104,142,162,163,164]. Several groups have also demonstrated the ability to use slot-die coating under ambient conditions, both on rigid [162,164] and flexible substrates [64,88,168]. Other areas of interest for slot-die-coated devices include methods for improving stability [63,88,94,104,162], the fabrication of indoor-based OPVs [64,67], using ITO-free substrates [113,166,168,169], the formation of a ternary active layer [163], device encapsulation [94], and alternative coating methods, from the sequential deposition of the active layer [163] to the use of differentially pumped slot-die coating [170].

With the versatility of slot-die coating and the ability to coat using a range of viscosities, there have been a wide range of materials coated with this technique, including but not limited to active layer blends (i.e., P3HT:PCBM [90,165], PPDT2FBT:PCBM [67,88], PBDB-T:ITIC [113], and PM6:Y6 [170,175]), electron (i.e., ZnO NPs [161,162,170], SnO_2_ [58,176], PEI [139], and AZO [85,153]) and hole transport layers (i.e., PEDOT:PSS [139,165]), and electrodes (i.e., AgNWs [176,177]). As for the number of layers coated via slot-die coating on a single device, a significant amount of papers have implemented this technique for two [155,162,178] or three layers [104,105,154,179], with a minor amount applying the technique for a single layer [42,65,87]. There are examples of fully printed devices that utilise slot-die coating, however, they usually use other printing methods such as flexography [161,180] or screen [64,180] printing for the electrodes.

Though not a popular as blade coating, there is still significant interest in the coating of high-performance active materials via slot-die coating, however, this is mostly limited to laboratory- and small-scale devices. As such, the highest efficiency achieved using this process to construct the active layer was 16.2% with an undisclosed active area (defined as laboratory scale by the author), using PM6:Y6 [181], while the largest area attempted was 360 cm^2^ with an efficiency of 1.18% using P3HT:PC61BM [90].

With the resurgence of interest in slot-die coating, primarily of the active layer, there have been investigations into ways to improve OPV device performances, from the selection of appropriate solvents and solvent additives [86], solvent post-annealing [173], and the implementation of materials more suitable for slot-die coating [65]. There is also interest in using more environmentally friendly solvents, with minimal losses in device performance [104]. One method that has seen increased interest for this technique is hot deposition, a method for controlling morphology and printing quality by heating up the slot-die printing head and/or substrate during deposition. As a common method for MRCs, it has been demonstrated to allow for coating without the use of high-boiling-point/low-vapour-pressure solvents, while also shown to be R2R compatible via a bench-sized R2R coater [63].

In summary, slot-die coating has been demonstrated as a viable method of active layer and interface layer coating, aimed towards the fabrication of flexible OPV cells and modules, especially when implemented in roll-to-roll fabrication. In terms of large scales, however, more interest in utilising high-performance active layer materials is required to demonstrate the effectiveness of the technique compared to alternative printing and coating methods.

#### 3.4.5. Spray Coating

**Figure 9:**
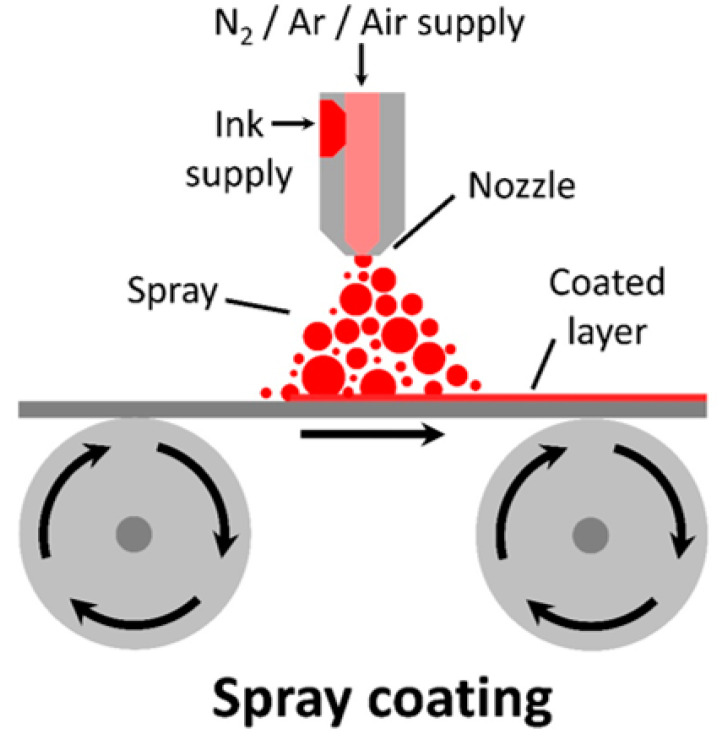
Basic schematic of spray coating. Adapted from Søndergaard et al., 2012 [135].

Spray coating is a contactless, zero-dimensional coating method where a continuous spray of ink is atomised into spray that is directed towards the substrate’s surface. By pushing the ink through a nozzle with the use of a nitrogen/argon/oxygen gas, ink is ejected to the free space and to the substrate (seen in Figure 9). The thickness can be adjusted by increasing the spray amount or reapplying layers.

To allow for a uniform coating during deposition, several papers have incorporated ultrasonic nozzles [182,183], allowing for a reduced droplet size without reducing the high pressure gas that would allow for large-area deposition [184]. Another method that has been demonstrated in the acoustic vibration of the substrate during deposition [182] is in situ annealing [183,185,186]. Spray coating has been used to process several active layer blend combinations (i.e., P3HT:PCBM [187,188,189,190]), electron transport layers (i.e., ZnO [188,191], PEIE [93]), hole transport layers (i.e., PEDOT:PSS [188,192], V_2_O_5_ [93]), and even AgNWs used as an electrode [191]. From an assessment of the literature, the majority of OPV devices fabricated via spray coating only used the technique to deposit the active layer [182,183,185,187,189,190,193,194], with the rest of the layers either being spin coated or deposited via vacuum-based processes. Very few papers have implemented spray coating for the film formation of two [182,195] or three layers [93]. It is also worth noting that the majority of devices using this technique were fabricated on a rigid substrate [183,186,189,192], with only one paper, to date, demonstrating deposition over a flexible substrate [189].

From 2010 to 2015, there was a lot of interest in this coating technique, especially towards improving the spray quality to allow for a uniform thin film. Since 2015, however, there has been less interest, while other coating techniques have gained popularity. As of 2021, the highest efficiency achieved using this process was 16% with 0.043 cm^2^, using PM6:N3 [196], while the large active layer attempted was in 2018 with P3HT:PCBM, having achieved 2.4% with an area of 4 cm^2^ [188]. Even though small-scale devices have been fabricated with spray coating [188,189], there has been very little research conducted to fabricate devices at medium or large scales. For spray coating, the areas that have been focused on, outside of the influence of the active layer, include ITO-free device fabrication [93,191,195], improving light degradation [192], producing all spray coated devices [188,191], and printing on uneven surfaces such as textiles [191]. Other areas also include the formation of bilayers in the active layer region by the implantation of “dry spray-coating” [197] and the use of s-MoO_3_ nanoparticle ink [70].

Even though there are a variety of implementations for spray coatings in OPV fabrication, they have not reached the same success as other non-contact coating methods, such as blade or slot-die coating. This can be attributed to the large amount of material wastage, resulting in groups using cheaper yet lower-performing materials to produce devices. Despite this, there is continued interest in this technique due to its versatility and ability to coat thin films on uneven surfaces. The technique has also been demonstrated to reach similar efficiencies as blade and slot-die coating when compared at the laboratory scale.

#### 3.4.6. Inkjet Printing

**Figure 10:**
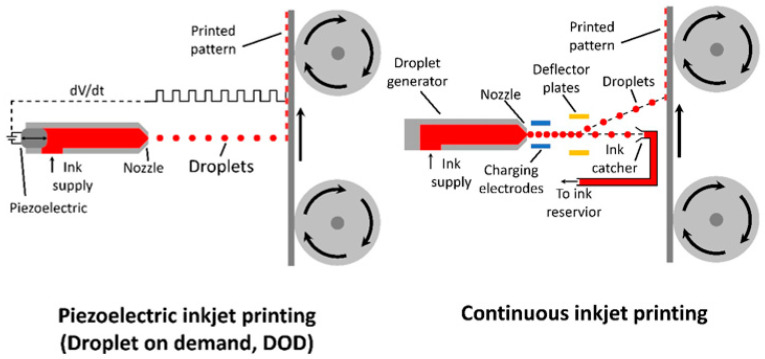
Basic schematic of two types of inkjet printing, piezoelectric (LEFT) and continuous (RIGHT). Adapted from Søndergaard et al., 2012 [135].

Inkjet printing is a two-dimensional printing method that can easily control the printed layer thickness and be adjusted by either re-coating over the same area or increasing the rate of ink deposition during printing. Unlike spray coating that covers a large area simultaneously, the ink produces ink droplets that follow a relatively linear path towards the substrate (seen in Figure 10), allowing for fine control of the coated area, as well as reducing ink wastage.

There are two main types of inkjets that have been employed for OPV fabrication: piezoelectric (a.k.a. droplet on demand, DOD) and continuous inkjet. For DOD inkjet printing, a piezoelectric-controlled pump is used to produce droplets when over a position that requires ink deposition, while the continuous inkjet has a continuous flow of ink drops, where the deposition is controlled with deflector plates, allowing the illusion of the inkjet stopping and starting [153].

The final dry thickness of inkjet-printed films (d) can be determined based on the number of droplets ($N_{d}$) delivered per area (cm^−2^), the individual droplet volume ($V_{d}$), and the concentration (C) and density (ρ) of the solid material in the ink, with the equation as follows (Equation (3)):

(3) $$d=N_{d}V_{d}\frac{c}{\rho }\;$$

Due to the ability to form patterns using printing, there is a relatively even distribution by way of using inkjet coating of the active layer, interface, and electrode deposition. Materials that have been coated using this technique include, but are not limited to, active layer materials (i.e., P3HT:PCBM [198,199], P3HT:O-IDTBR [200], and P3HT:IC_60_BA [143]), transport/interface materials, such as PEDOT:PSS [143,201] and ZnO [143,198], and electrodes, including Ag [202,203] and Ag NWs [204]. The majority of papers utilise inkjet printing for multiple layers on a single device, either for two [198,205,206] or three [198,201] layers, with exceptions for active layers [143,207] or electrode-only printing [203,208], as well as fully printed devices [143,200,204].

Though there has been some interest in employing inkjet coating for high-performance OPVs, reaching up to 7.3% PCE using p-DTS(FBTTh_2_)_2_:PC_71_BM [207], P3HT:PCBM blend devices still dominate the number of devices fabricated with this method [198,199,205]. The highest efficiency with an upscaled active area of 1 cm^2^ reached 4.7% using active material “Activlink PV2000” [201].

Due to the printing web speed, it will be difficult for the technique to compete with faster printing and coating methods, especially when coating the active layer; however, there is still interest in high-resolution printing, especially for sheet-to-sheet device fabrication, as well as laboratory-scaled printing, where minimal wastage is a necessary requirement for reducing manufacturing costs.

#### 3.4.7. Pad Printing

**Figure 11:**
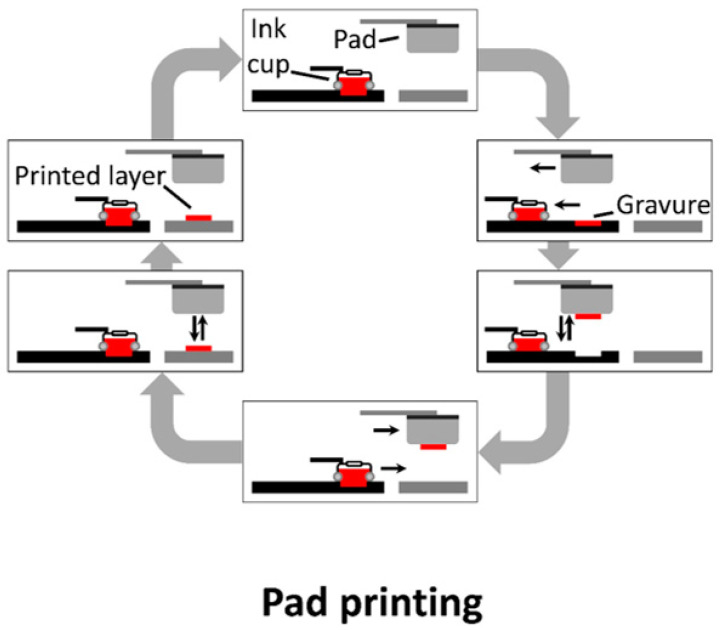
Basic schematic of pad printing. Adapted from Krebs et al., 2009 [209].

Pad printing is a two-dimensional, contact-based printing method that implements a pad to collect ink from a gravure and transfers it to a substrate. As seen in Figure 11, a gravure is filled from the ink cup with ink, prior to a pad picking up the ink and transferring it to a substrate.

The final dry thickness of the deposited film can be calculated based on the volume of ink contained in the gravure per unit area ($V_{g}$) (cm^3^ m^−2^), the concentration of solid material in the printing ink (c), and the material density of the dry film (ρ) (seen in Equation (4)):

(4) $$d=V_{g}k_{p}\frac{c}{\rho }\;$$

It is worth noting that the pickup and deposition of the ink from the pad are not always complete, resulting in a reduction in the expected dry film thickness. As such, a constant ($k_{p}$) is associated with the thickness equation. So far, there has only been one paper to demonstrate pad printing for the fabrication of OPV devices, with the results published by Krebs et al., 2009, with the printing of the P3MHOCT + Zinc resulting in an efficiency of 0.07% [209]. Due to the complexity of the printing method instrumentation, the pad coating method has seen little interest for all scales of OPV fabrication, especially with the existence of better printing and coating systems that are available on the market.

#### 3.4.8. Gravure Printing

**Figure 12:**
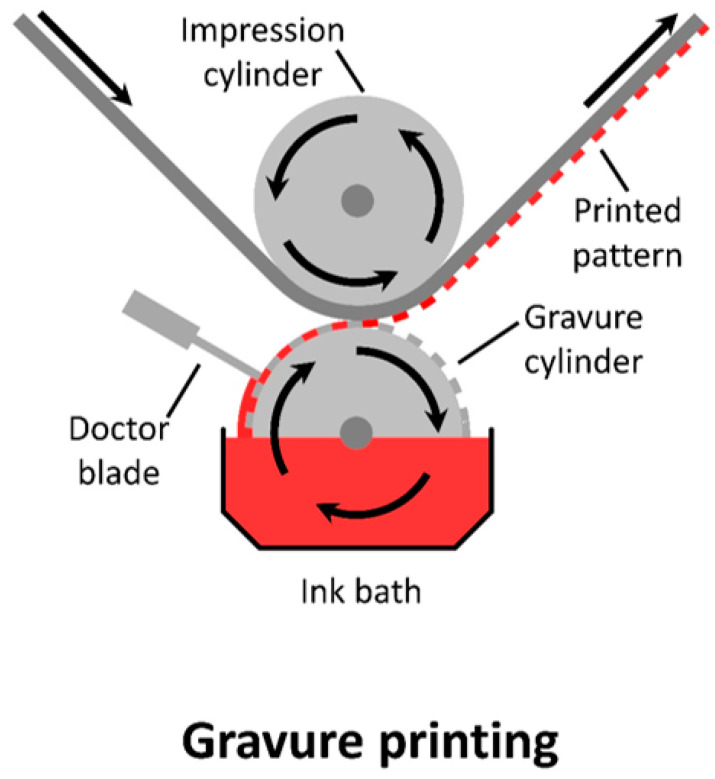
Basic schematic of gravure printing. Adapted from Søndergaard et al., 2012 [135].

Gravure is a contact-based, two-dimensional coating method, with the pattern forming from the ink within the cavities of the engravings. The basic version of this printing method has the ink being withdrawn from an ink bath, with any excess ink being removed via a blade. A substrate is then fed between the impression cylinder and the ink-covered gravure roll to imprint a patterned coating (seen in Figure 12). As such, this technique relies on the surface energy of the substrate to remove the ink from the cavities as the web is brought into contact with the cylinder.

Both the thickness and shape of the printed layer are dependent on the engraved pattern and depth of the cavities of the cylinder, making it difficult and costly to optimise the coating thickness, however, some papers have shown that adjustment of the blade against the gravure cylinder can influence the amount of ink deposited [210,211,212,213]. The quality of printing is dependent on the ink rheology, web/substrate speed, and pressure between the impression cylinder and gravure cylinder against the cylinder.

For the past 10 years, gravure printing has been demonstrated to be compatible for the roll-to-roll printing of OPVs [212,213,214], having printed transport layers such as ZnO, ZnO NP or ZnO:PEI [211,214], PEDOT:PSS [151,212,213], as well as being used in active layer printing. The initial work was based on P3HT:PCBM devices [210], however, this has expanded to more moderately performing materials, such as PTB7-Th:PC71BM devices [214]. Despite these limiting factors involved in the technique itself, there have been several attempts at fabricating devices with gravure as the major printing method. As such, the highest-performing device achieved by gravure was 6.6% PCE, using PTB7-Th:PC_71_BM and having an active area of 0.09 cm^2^ [214], while the largest-area device was 75 cm^2^, achieving a PCE of 0.86% when using P3HT:PCBM [213].

Though there was a lot of interest in the 2010s, there has been less research focused on improving this method, generally due to the amount of material used in the method. As such, devices mostly used P3HT:PCBM as an active material for OPVs fabricated with gravure printing. Despite this method being roll-to-roll compatible, the technique is difficult to downscale and there is a large amount of material waste, making it less desirable for lab-scale device fabrication. It is also worth noting that the method has seen more devices made with flexible substrates [151,210,211,212], with very few performed (if any) on rigid substrates. Some work that has been focused on gravure-printed devices includes optimising the plate and grid sizing, improving the mechanical stability [211,212], and optimising conditions to reduce the gap between gravure and spin-coated devices [209,211].

Based on the number of papers, gravure peaked in interest for the printing of the active layer around 2017 [214,215], however, this interest has waned as of late. Several factors, including its difficulty of use in laboratory-scale fabrication, potential material wastage, and more focus on blade- and slot-die-coated active layer research, have made this method less desirable for OPV research of high-performance devices. Despite this, there has still been interest in the gravure printing of ZnO:PEI and ZnO as the bottom interface for inverted OPVs [210,211,216].

#### 3.4.9. Flexographic Printing

**Figure 13:**
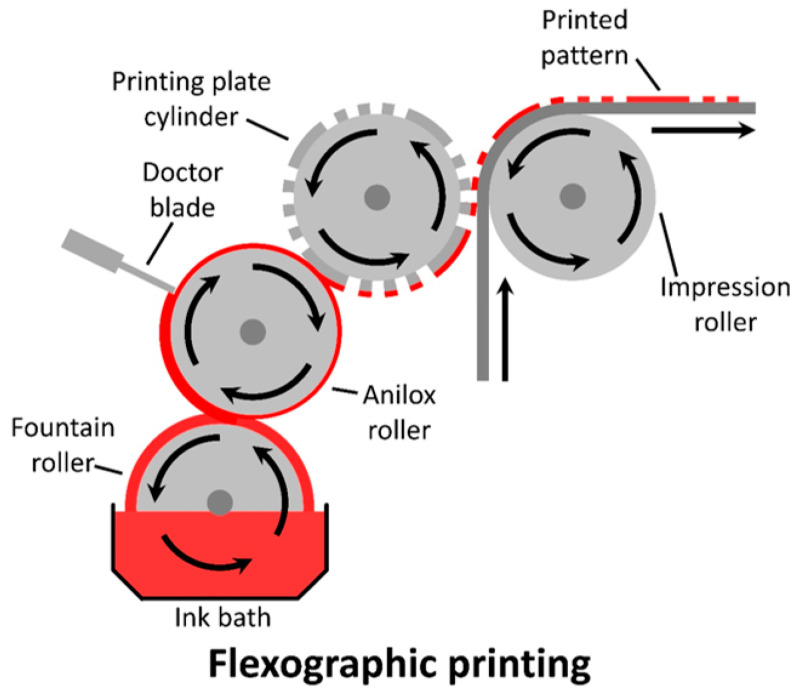
Basic schematic of flexographic printing. Adapted from Søndergaard et al., 2012 [135].

Flexographic printing (also known as flexography) is another two-dimensional, contact-based printing method that involves direct contact with the substrate. Unlike gravure printing, which relies on filling engravings with ink, flexographic printing has the ink being deposited from patterned plates from the cylinder. To achieve this via roll-to roll fabrication, a foundation collects ink from an ink bath and deposits it onto an anilox roll. To control the wet thickness, a doctor blade can be used. The ink is then transferred onto the printing plate cylinder before being deposited onto a substrate that is pressed against the printing plate and impression roll (seen in Figure 13). This method can be downscaled for implementation on a mini-roll coater, with the mini-roll acting as the impression roll, while the deposition of the ink onto the printing plate cylinder occurs manually [168,217].

Based on the properties that flexographic printing relies on, this method allows for the printing of inks that have a higher viscosity, unlike gravure, which relies on low viscous inks. This has allowed this printing method to be commonly used for the printing of electrodes of ITO-free OPVs [82], with the most common material being Ag-based paints [114,168,169,208,218]. This technique has also been used for the printing of the electron transport layer (ZnO) and active layer containing PCDTBT:PC71BM [82]. To date, the highest efficiency achieved with the flexographic printing of the active layer was 3.4%, using PCDTBT:PC71BM as the active material and having an active area of 1 cm^2^ [82].

Other research that has been conducted for this technique includes improving printing by improving pre-wetting techniques [114], patterning optimisation to improve resolution [219,220], and adjusting the ink preparation and printing temperature [221]. However, a lot of this research is aimed at flexible electronic devices, rather than focusing on OPV device fabrication.

Like gravure printing, due to the large amount of ink wastage, complexity, and the difficulty in performing laboratory-scale fabrication, there has been minimal amount of interest from the OPV community in investigating this technique. Despite this, the method is still used for scalable ITO-free OPV device fabrication.

#### 3.4.10. Screen Printing

**Figure 14:**
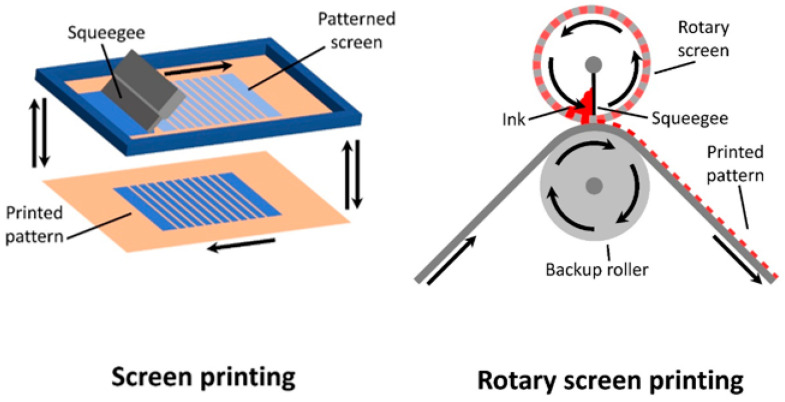
Basic schematic of screen printing, specifically flatbed and rotary. Adapted from Søndergaard et al., 2012 [135].

Screen printing is a contact-based, two-dimensional printing method where ink is forced through a patterned screen with the use of a squeegee, resulting in the ink being deposited onto the waiting substrate below (seen in Figure 14).

Unlike flexographic and gravure printing, screen printing allows for the formation of thicker films, making it ideal for printing of electrodes. Currently, there are two major methods of screen printing implemented in OPV fabrication, flatbed (laboratory scale) and rotary (large scale). For the flatbed, ink is based over a flat, patterned screen, allowing for the sheet-to-sheet patterning of flat substrates [222,223,224]. As for rotary, the patterned screen is shaped into a roll, with the squeegee and ink positioned inside, making it ideal for roll-to-roll device fabrication [114,180].

The final dry thickness of the film (d) can be determined based on the theoretical volume of ink contained within the screen ($V_{Screen}$), the pick-out ratio ($k_{p}$), the concentration of the solid material in the ink (c), and the dry film density (ρ) (seen in Equation (5)):

(5) $$d=V_{Screen}k_{p}\frac{c}{\rho }$$

There is not much research that primarily focuses on the application of screen printing, with that that does having printed PEDOT:PSS over the active layer [223], the active layer only [225], or the Ag grid on top of ITO and the active layer [222]. For devices with an active layer deposited via screen printing, the highest efficiency achieved was 2.4% with an active area of 3 cm^2^, having an active material of P3HT:PCBM [225].

There has been little investigation into screen printing as a method for active layer coating, however, it has become one of the most common methods for the scalable and upscale deposition of electrodes, for both the top [114] and bottom [180,222,226], as well as the transport layers [166,180,223]. Screen printing is also commonly used for depositing an etching solution over ITO-covered PET, allowing for the patterning of the substrate prior to device fabrication [82,114,150,159]. With the existence of both flatbed and rotary screen printing techniques, this has allowed for screen printing to be demonstrated to deposit films over rigid [180,225] and flexible substrates [222,224].

Despite the lack of interest in active layer deposition using screen printing, the technique has still found use for patterned electrode printing, for both flatbed and rotatory screens. This has allowed for the technique to remain relevant, especially for device fabrication that is moving away from vacuum-based processes.

### 3.5. Printing and Coating Method Comparison

As seen so far in this chapter, there is quite an extensive array of printing and coating techniques that have been demonstrated to work successfully towards the fabrication of OPVs, however, some techniques have seen more success in publication numbers than others due to a multitude of factors. For an in-depth comparison of the printing and coatings techniques, Table 1 was prepared, comparing the device architectures, printing/coating methods implemented, active areas, and device performances. This section will also relate the summary of the literature with the technical comparison of printing and coating techniques (seen in Table 2). It can be observed that some techniques, such as blade and slot-die coating, have seen extensive use for active layer deposition, while gravure inkjet printing and spray coating have only experienced moderate interest. As for rod coating and rod printing, they have seen minimal interest for OPV fabrication. Lastly, though flexography and screen printing have seen little interest for active layer deposition, they have been used extensively for PEDOT:PSS and Ag electrode printing (Table 2), adapted from Krebs et al., 2009 [119].

It can be observed that some techniques, such as blade and slot-die coating, have seen extensive use for active layer deposition, while gravure inkjet printing and spray coating have only experienced moderate interest. As for rod coating and rod printing, they have seen minimal interest for OPV fabrication. Lastly, though flexography and screen printing have seen little interest for active layer deposition, they have been used extensively for PEDOT:PSS and Ag electrode printing.

Ink preparation (focused on controlling the physical properties of the printed thin film) is relatively simple for spin, blade, slot-die, and spray coating. By contrast, for pad, gravure, flexography, and screen printing, the range of printable inks is more limited. This can be an issue for active layer deposition, since ink preparation strongly influences BHJ morphology [159,175,243,244].

**Figure 15:**
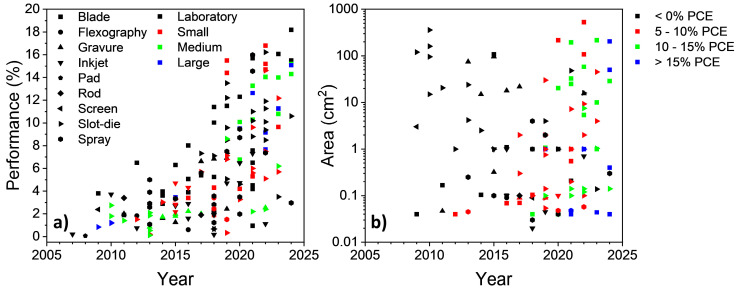
(**a**) Performance vs. year, and (**b**) active area vs. year of device fabrication, with the grouping primarily based active layer coating. Data points are based on the values from Table 1.

When plotting PCE in relation to year of publication (Figure 15a), it can be observed that there has been a significant improvement in performance since 2010.

PCEs for blade-coated devices have reached as high as 18.2%, whereas the highest slot-die coated devices are somewhat lower, with a PCE of 16.2%. On the other hand, devices with active layers coated with spray, inkjet, or gravure printing have not reached above an 8% PCE. This increase in PCE can be attributed primarily to the development of high-performing materials, with spin-coated devices reaching over 19% since 2022 [13,15].

Although there has been a steady increase in the PCE being achieved by alternate printing and coating techniques, the fabrication and testing of OPV devices are dominated by devices below 1 cm^2^, as shown by the plot of active layer area in relation to year of publication (Figure 15b). In early times, slot-die coating took up the majority of large active area device fabrication due to their promising ability to fabricate flexible devices. As other printing and coating methods were being investigated, gravure gained interest for the fabrication of larger-area devices from 2014 to 2017. Since 2017, blade coating has been responsible for the majority of large-scale fabricated devices, due to its ease-of-use as a coating method, as well as its ability to coat rigid substrates.

**Figure 16:**
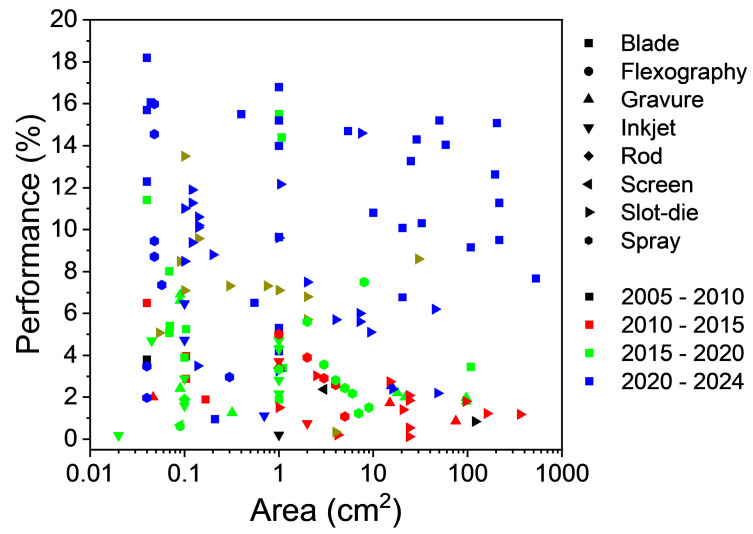
Performance vs. active area where the active layer with the grouping primarily based active layer coating. Data points are based on the values from Table 1.

Although majority of OPV fabrication has been focused on laboratory-scale (<1 cm^2^) devices, there has been growing interest in the fabrication of high-performance (>10% PCE) devices with medium and large active areas. Considering PCE as a function of active area (Figure 16), it is observed that blade-coated devices are amongst the highest-performance OPVs fabricated across all scales, with the highest PCE reaching 18.2% (active area non-disclosed), whereas a PCE of 15.08% was achieved with an active area of 204 cm^2^. Despite the 17% reduction in PCE with an increase in the active area, this result demonstrates that blade coating does have the ability to be upscaled for rigid-based devices at the cost of a small reduction in performance.

As for slot-die coating, the highest performance reached was 16.2% (non-disclosed active area), while the largest area achieved was 360 cm^2^ (1.18%). This gap is related to the difference in materials used between the two device sets, with 13.5% PCE devices having PTB7-Th:PC_71_BM:COi8DFIC in the active layer, while the 1.18% device used P3HT:PCBM. Since 2014, there has been a significant drop in interest in developing the technique for medium- and small-scale devices, coinciding with the rise of blade-coated devices. Despite this reduction, there has been a recent resurgence of slot-die coated OPVs at the small and medium scale. The case for slot-die coated also highlights the importance of comparing devices with the same active layer, as the choice of active materials plays a significant role in dictating the device performance. As such, this should be considered when comparing devices that use the same, if not similar, active materials to investigate the feasibility of printing and coating techniques. Despite this, there is a performance gap between spin-coated and slot-die-coated devices at the laboratory and small scale.

**Figure 17:**
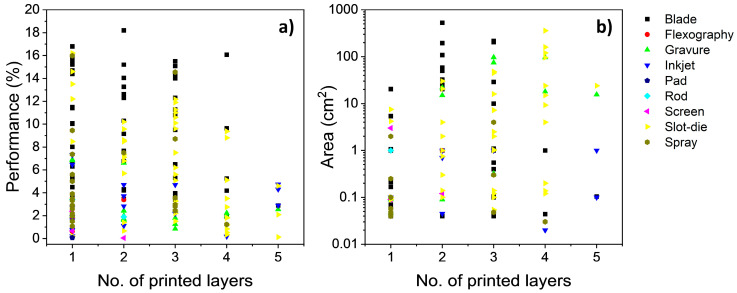
(**a**) Performance vs. number of printed layers, and (**b**) active area vs. number of printed layers, where number of coated layers is based on layers that have been applied via solvent-based techniques (excluding spin coating), with the grouping primarily based on active layer coating. Data points are based on the values from Table 1.

Another aspect of device fabrication that can be analysed is the number of layers that were printed/coated using solvent-based (excluding spin coating) deposition techniques. For simplicity, the devices will be named number-layered devices, indicating the number of layers coated with an alternative printing/coating technique (i.e., one-layered devices indicate only one layer was coated with a method that was not spin coating or vacuum-based). When observing Figure 17a,b, it can be found that one-layered devices have been predominantly been prepared as laboratory-scaled devices, with some exceptions for the small and medium scale.

The exact purpose of this is related to allowing for the optimisation of a single coating condition, as well as to investigating the feasibility of unique materials for active layers, interfacial layers, or electrodes. An example of this can be observed with the highest-performing blade-coated device that achieved a PCE of 15.7% with an active area of 0.04 cm^2^.

By contrast, scalable devices (small, medium, and large devices) are dominated by two- and three-layered devices. As mentioned previously, spin coating has issues with an increased area due to an uneven film thickness during deposition [114,119]. As such, it is important that the bottom interface layer, usually either PEDOT:PSS [84,137] (conventional devices) or ZnO [81,140] (inverted devices), is relatively uniform before depositing the active layer. The interfacial layer can then either be deposited via solvent-based processing (i.e., ZnO NPs [201], PEDOT:PSS [143]) or via vacuum-based deposition (i.e., MoOX [198], LiF [208]). Lastly, the electrode is deposited via a vacuum-based process. These types of devices are the most desirable type, as they enable the scalable fabrication (>1 cm^2^) of OPVs without sacrificing performance too much. This can be observed with a large-scale blade-coated device that reached 12.63% with an active area of 194.8 cm^2^, having blade coated the bottom interface and active layer [81]. Though blade coating was only used for two layers, with vacuum-based deposition of the top interface and electrode layer, this not an issue with rigid substrates, as they follow a sheet-to-sheet fabrication process. Three-layered devices have also been reached with blade coating, reaching a PCE of 9.5% [137].

As for four and five-layered devices, there appears to be limited interested in their development in terms of published work. In terms of four-layered devices, they require, excluding the bottom electrode, to be deposited via solvent-based processes. With a few exceptions, such as AgNWs [141,203], they are predominantly deposited via flexographic [218] or screen printing [138]. Due to the increases in ink wastage (seen Table 2) and technique complexity compared to blade, slot-die, and blade coating, there is less motivation for the OPV community to utilise these techniques. There is a further interest in these techniques for top electrode deposition due to the development of large-scale and roll-to-roll vacuum deposition systems. Despite these drawbacks, the techniques do allow for a cheaper method for printing patterns electrodes. In 2010, slot-die, in combination with flexographic and screen printing, demonstrated that a combination of printing coating techniques can be used to prepare large-area devices, however, due to the use of low-performance materials (P3HT:PC_61_BM), the performance was limited to 1.79% [90]. Though the majority of four-layer devices have occurred with a slot-die-coated active layer, the majority of these devices were fabricated before 2015 using low-performance materials [90,180,236].

As for five-layered devices, they have the added challenge of replacing the ITO layer, which acts as a transparent bottom electrode. Though alternative electrodes have been developed, such as Ag/PEDOT:PSS [173,180] and AgNWs/PEDOT:PSS [141], they do not appear to reach similar performances compared to ITO-based devices. Despite this issue, five-layered devices have been shown to work at a medium scale, with slot-die-based devices reaching a PCE of 2.09% with an area of 24 cm^2^. Overall, four and five-layered devices, though having the potential for the fabrication of relatively cheap OPV devices, they are less appetising for the OPV community to pursue due to their increased ink wastage and technique complexity.

Lastly, when reading through Table 1, many papers investigating the performance of printed and/or coated OPVs primarily aim at developing improved photoactive or interfacial materials. There have been few papers that aim at improving the printing technique themselves, either during or post deposition. Such techniques including the heating of the substrate/printing head [63,84], use of air/nitrogen to improve coating uniformity [137], and influencing the printing quality by the choice of solvent, combination, and additives, which all have an impact on influencing the printing quality [41,175]. This is especially true with blade coating, where the combination of in situ annealing [84,137,140,141,145,151,152,153] and the use of hot airflow [84,137] allowed for large-area and high-performing OPVs.

From this paper, it was found that blade coating is ideal for coating the active layer on rigid substrates, while slot-die coating demonstrated its ability for coating on flexible substrates. Though less interest has been shown in spray coating, past work has shown that the technique is viable for coating over rough/uneven substrates, including fabric- and fibre-based surfaces. As for inkjet printing, while the technique is not ideal for large-area devices, it is still useful to investigate for micro-sized devices due to its ability to form 3D patterns without the use of laser etching or lithography. Lastly, contact-based printing/coating techniques may not be ideal for active layer printing due to ink wastage and complexity, however, these techniques have been proven viable for the printing/coating of interfacial and electrode layers.

Overall, each printing condition has its advantages and disadvantages (highlighted in Table 3). With the advent of upscaling coating, the majority have been based on blade and slot-die coating, with a constant stream of spray-based coatings. Since 2019, there has been a spike in papers around working module devices, usually consisting of several cells on one substrate tested altogether. With all the methods having advantages and disadvantages, it is unlikely that a single printing/coating method can be used to produce high-performance OPVs, instead, it is likely that a combination of coating methods will be used. There have also been examples of roll-to-roll setups that implement several different coating instruments in a single fabrication line.

Overall, there are some groups that use blade coating to demonstrate upscaled printing, while other groups use blade coating as a step towards upscale roll-to-roll, usually then performing either roll-to-roll or MRC slot-die coating [139], while others are using blade coating as the first step away from relying on spin coating as a fabrication process. Blade coating is also a viable coating method for scalable OPV fabrication for rigid substrates via a flatbed. This technique seems to struggle for roll-to-roll fabrication methods.

## 4. Outlook

Overall, there has been considerable progress towards the improvement in OPV performance, reducing the gap between OPVs and silicon-based PVs. There has also been an increased focus on developing techniques for the fabrication of devices utilising printing and coating techniques that transition from laboratory-scale towards industry-scale devices.

When investigating the research that has been conducted for scalable and large-scale OPVs, most of the focus is on reducing the performance gap between laboratory-scale spin-coated devices and alternatively printed/coated OPVs. As such, only a small amount of the literature has focused on determining scalable methods for improving the lifespan of said devices. On the positive side, in the past 5 years, there has been a shift in laboratory-scaled area printing and coating under nitrogen conditions towards more scalable fabrication under ambient conditions, allowing for research to more closely follow the deposition procedures used in upscaled manufacturing.

Despite the shift to alternative printing and coating methods, and the continued increases in performance that can be achieved with OPVs, there are still few efforts directed towards improving the performance of larger (>1 cm^2^) active area devices. For OPV scalable fabrication to be more competitive in the PV field, there needs to be more focus on scalable fabrication methods that bridge between lab-scale and large-scale fabrication processes and investigations of strategies for improving the stability of scalable and large-scale devices. This focus on research on scalable OPV fabrication would allow for better connections between laboratory-scale and large-scale research, accelerating the field towards commercialisation.

## Figures and Tables

**Table 1:** List of printing and coatings techniques, including device architecture, printed layers, device area, and device performance. Cells/modules with an area > 1 cm^2^ with a power conversion efficiency > 5% are highlighted in bold.

Method	Year	Device Structure	Printed Layers	Area (cm^2^)	Performance (%)	Cell or Module	Ref.
Blade	2009	Glass/ITO/PEDOT:PSS/P3HT:PCBM/Ca/Al	P3HT:PCBM (Blade)	0.04	3.8	Cell	[101]
	2011	Glass/ITO/PEDOT:PSS/P3HT:PCBM/LiF/Al	P3HT:PCBM (Blade)	0.167	1.9	Cell	[161]
	2012	Glass/ITO/PEDOT:PSS/POD2T-DTBT:PC_71_BM/Al	POD2T-DTBT:PC_71_BM (Blade)	0.04	6.49	Cell	[102]
	2014	Glass/ITO/PEDOT:PSS/pDPP5T-2:PC61BM/ZnO NPs/Ag	PEDOT:PSS, pDPP5T-2:PC_61_BM and ZnO NPs (Blade)	0.104	3.96	Cell	[141]
	2014	Glass/AgNW/PEDOT:PSS/pDPP5T-2:PC_61_BM/ZnO NP/AgNW	AgNW, PEDOT:PSS, pDPP5T-2:PC_61_BM and ZnO NPs, AgNW (Blade)	0.104	2.89	Cell	[141]
	2014	Glass/ITO/PEDOT:PSS/PTB7:PC_61_BM/Ca/Al	PTB7:PC_61_BM (Blade)	-	3.61	Cell	[151]
	2015	Glass/ITO/ZnO/pDPP5T-2:PC_71_BM/MoO_3_/Ag	pDPP5T-2:PC_71_BM (Blade)	-	6.3	Cell	[146]
	2015	Glass/ITO/PEDOT:PSS/POD2T-DTBT:PC_71_BM/LiF/Al	PEDOT:PSS, POD2T-DTBT:PC_71_BM (Blade)	108	3.45	Module	[84]
	2016	Glass/ITO/ZnO/PBDT-TSR:PPDIODT/MoOx/Al	PBDT-TSR:PPDIODT (Blade)	0.069	5.07	Cell	[145]
	2016	Glass/ITO/ZnO/PBDT-TSR:PC_71_BM/MoO_X_/Al	PBDT-TSR:PC_71_BM (Blade)	0.069	8.02	Cell	[145]
	2016	Glass/ITO/ZnO/P3HT:IC_60_BA/PEDOT:PSS/Ag	ZnO, P3HT:IC_60_BA and PEDOT:PSS (Blade)	0.1	3.9	Cell	[143]
	2016	Glass/ITO/ZnO/P3HT:IC_60_BA/PEDOT:PSS/Ag	ZnO, P3HT:IC_60_BA and PEDOT:PSS (Blade)	1.1	3.4	Cell	[143]
	2017	Glass/ITO/ZnO/P (T3-TPD):PC_71_BM/MoO_3_/Ag	P(T3-TPD):PC_71_BM (Blade)	0.07	5.40	Cell	[85]
	2018	PET/ITO/ZnO/PCDTBT:PC_71_BM/MoO_X_/Ag	ZnO and PCDTBT:PC_71_BM (Blade)	1	4.3	Cell	[82]
	2018	PET/ITO/ZnO/PCDTBT:PC_71_BM/MoO_X_/Ag	ZnO, PCDTBT:PC71BM and VO_X_ (Blade). Ag (Flexography)	1	1.9	Cell	[82]
	2018	ITO/ZnO/PBDB-T:ITIC/MoOx/Al	PBDB-T:ITIC (Blade)	-	10.03	Cell	[123]
Blade	2018	Glass/ZnO NP/P3HT:IDTBR/PEDOT:PSS/Ag NWs	ZnO NP, P3HT:IDTBR, PEDOT:PSS and Ag NWs (Blade)	0.1 04	5.25	Cell	[144]
	2018	Glass/ITO/ZnO/PBTA-TF:IT-M/MoO_3_/Al	PBTA-TF:IT-M (Blade)	0.04	11.4	Cell	[149]
	2019	Glass/ITO/ZnO/PBDB-T:iIEICO-4F/MoO_3_/Al	PBDB-T:iIEICO-4F (Blade)	-	11.5	Cell	[65]
	2019	Glass/PEDOT:PSS/PBDB-TF:BTP-4Cl-12/PDINO/Al	PBDB-TF:BTP-4Cl-12 (Blade)	1.0	15.5	Cell	[152]
	**2019**	**Glass/ITO/PEDOT:PSS/PBDB-TF-T1:BTP-4F-8/PFN-Br/Al**	**PBDB-TF-T1:BTP-4F-8 (Blade)**	**1.07**	**14.4**	**Cell**	[147]
	2020	PET/ITO/ZnO/PDTSTPD:PC_71_BM/MoO_X_/Ag	ZnO and PDTSTPD:PC_71_BM (Blade)	1	4.2	Cell	[159]
	2020	Glass/ITO/ZnO/PCDTBT:PC71BM/MoO_3_/Ag	PCDTBT:PC_71_BM and ZnO (Blade)	0.04	12.3	Cell	[157]
	**2020**	**glass/ITO/ZnO/TPD-3F:IT-4F/MoO_X_/Ag**	**TPD-3F:IT-4F (Blade)**	**20.4**	**10.08**	**Module**	[148]
	**2020**	**glass/ITO/ZnO/TPD-3F:IT-4F/m-PEDOT:PSS/Ag**	**PEDOT:PSS and TPD-3F:IT-4F (Blade)**	**20.4**	**6.77**	**Module**	[148]
	2020	Glass/ITO/PEDOT:PSS/NF3000-P:NF3000-N/TASiW-12/Al	PEDOT:PSS,NF3000-P:NF3000-N and TASiW-12 (Blade)	0.04	12.3	Cell	[137]
	**2020**	**Glass/ITO/PEDOT:PSS/NF3000-P:NF3000-N/TASiW-12/Al**	**PEDOT:PSS,NF3000-P:NF3000-N and TASiW-12 (Blade)**	**216**	**9.5**	**Module**	[137]
	**2021**	**Glass/ITO/ZnO/PM6:Y6:PC_61_BM/MoO_X_/Ag**	**ZnO and PM6:Y6:PC_61_BM (Blade)**	**25**	**13.27**	**Module**	[81]
	**2021**	**Glass/ITO/ZnO/PM6:Y6:PC_61_BM/MoO_X_/Ag**	**ZnO and PM6:Y6:PC_61_BM (Blade)**	**194.8**	**12.63**	**Module**	[81]
	2021	PET/ITO/ZnO NPs + PEIE/P3HT:PC_61_BM/PEDOT:PSS/Ag	Ag top (Blade)	0.21	0.95	Cell	[158]
	2021	PET/ITO/Ag/ITO/PEI/RaynergyTek:PC_61_BM/PEDOT:PSS/Ag	PEI, RaynergyTek:PC_61_BM and PEDOT:PSS (Blade)	0.55	6.5	Cell	[139]
	2021	PET/ITO/Ag/ITO/PEI/C1:PC_61_BM/PEDOT:PSS/Ag	C1:PC_61_BM, PEI, PEDOT:PSS (Blade)	0.55	6.5	Cell	[139]
	**2021**	**PET/ITO/ZnO NPs/PCDTBT:PC_71_BM/MoO_3_/Ag**	**Pattern ITO (Screen). ZnO NPs and PCDTBT:PC_71_BM (Blade)**	**1**	**5.3**	**Cell**	[150]
	2021	Glass/ITO/ZnO/PTB7-Th:P(NDI2OD-2T)/MoO_3_/Ag	PTB7-Th:P(NDI2OD-2T) (Blade)	-	4.5	Cell	[150]
	2021	Glass/ITO/ZnO/PV-X Plus/MoO_3_/Ag	PV-X Plus (Blade)	0.04	15.7	Cell	[140]
	**2021**	**Glass/ITO/ZnO/PV-X Plus/MoO_3_/Ag**	**ZnO, PV-X Plus (Blade)**	**32.64**	**10.3**	**Module**	[140]
	2021	Glass/ITO/ZnO/PTB7-Th:PC_71_BM/MoO_3_/Al	ZnO and PTB7-Th:PC_71_BM (Blade)	-	7.58	Cell	[70]
	2021	Glass/ITO/ZnO/PTB7-Th:PC_71_BM/MoO_3_/Ag NWs	ZnO and PTB7-Th:PC_71_BM (Blade). MoO_3_ and Ag NWs (Spray)	-	4.19	Cell	[70]
Blade	**2022**	**PET/Ag/ZnO/ZnO NPs/PM6:Y6:PC_71_BM/MoO_X_/Ag**	**ZnO NPs, PM6:Y6:PC_71_BM (blade)**	**108**	**9.15**	**Module**	[227]
	**2022**	**PET/Ag/ZnO/ZnO NPs/PM6:Y6:PC_71_BM/MoO_X_/Ag**	**ZnO NPs, PM6:Y6:PC_71_BM (blade)**	**528.5**	**7.67**	**Module**	[227]
	**2022**	**Glass/ITO/ZnO/PBDB-T-2F:N3:P(NDI2OD-T2)/MoO_X_/Ag**	**PBDB-T-2F:N3:P(NDI2OD-T2) (Blade)**	**58.5**	**14.04**	**Module**	[228]
	**2022**	**Glass/ITO/PEDOT:PSS/PM6:T8/PNDIT-F3N/Ag**	**PM6:T8 (Blade)**	**1**	**16.8**	**Cell**	[42]
	**2022**	**Glass/ITO/ZnO/PM6:N3:PY-P2/MoO_3_/Ag**	**PM6:N3:PY-P2 (Blade)**	**1**	**15.2**	**Cell**	[177]
	**2022**	**Glass/ITO/ZnO/PM6:N3:PY-P2/MoO_3_/Ag**	**PM6:N3:PY-P2 (Blade)**	**5.4**	**14.7**	**Module**	[177]
	2023	Glass/ITO/PEDOT:PSS/PM6:BTP-eC9/PNTDIT-F3N/Field’s metal	PEDOT:PSS, PM6:BTP-eC9, PNTDIT-F3N, Field’s metal (Blade)	0.044	16.07	Cell	[229]
	**2023**	**Glass/ITO/PEDOT:PSS/PM6:BTP-eC9/PNTDIT-F3N/Field’s metal**	**PEDOT:PSS, PM6:BTP-eC9, PNTDIT-F3N, Field’s metal (Blade)**	**1**	**9.64**	**Cell**	[229]
	**2023**	**Glass/ITO/ZnO/PEI/PM6:Y6/MoO_3_/Ag**	**ZnO, PEI, PM6:Y6 (Blade)**	**216**	**11.27**	**Module**	[156]
	**2023**	**Glass/ITO/PEDOT:PSS/PM6:Y6:PC_71_BM/BCP/Ag**	**PEDOT:PSS, PM6:Y6:PC_71_BM, BCP (Blade)**	**1**	**14**	**Cell**	[154]
	**2023**	**Glass/ITO/PEDOT:PSS/PM6:Y6:PC_71_BM/BCP/Ag**	**PEDOT:PSS, PM6:Y6:PC_71_BM, BCP (Blade)**	**10**	**10.8**	**Module**	[154]
	**2024**	**ITO/ITO/PEDOT: PSS/PBQx-TF: TBT-26/PDINN/Ag**	**PEDOT:PSS (Rod). PBQx-TF: TBT-26, PDINN (Blade)**	**28.8**	**14.3**	**Module**	[155]
	2024	Glass/ITO/ZnO/PM6:Y6-C12:PC_61_BM/PEDOT-F/Ag	ZnO, PM6:Y6-C12:PC_61_BM, PEDOT-F (Blade)	0.4	15.5	Cell	[16]
	**2024**	**Glass/ITO/ZnO/PM6:Y6-C12:PC_61_BM/PEDOT-F/Ag**	**ZnO, PM6:Y6-C12:PC_61_BM, PEDOT-F (Blade)**	**204**	**15.08**	**Module**	[16]
	2024	Glass/ITO/ZnO/PB2:FTCC-Br:BTP-eC9/MoO_X_/Ag	ZnO, PB2:FTCC-Br:BTP-eC9 (Blade)	0.04	18.2	Cell	[230]
	**2024**	**Glass/ITO/ZnO/PB2:FTCC-Br:BTP-eC9/MoO_X_/Ag**	**ZnO, PB2:FTCC-Br:BTP-eC9 (Blade)**	**50**	**15.2**	**Module**	[230]
Flexography	2012	PET/Ag-grid/PEDOT:PSS/ZnO/P3HT:PCBM/PEDOT:PSS/Ag	Bottom Ag-grid (Flexography)	-	1.82	Cell	[208]
	2016	PET/Ag grids or (Ag grid/CNT hybrid coating)/PEDOT:PSS/P3HT:PC_61_BM/Ca/Al	Ag-grid (Flexography)	0.09	0.61	Cell	[218]
	2018	PET/ITO/ZnO/PCDTBT:PC_71_BM/MoO_X_/Ag	ZnO and PCDTBT:PC_71_BM (Flexography)	1	3.4	Cell	[82]
Gravure	2011	PET/PEDOT:PSS/P3HT:PC_61_BM/Ca/Al	PEDOT:PSS (Gravure)	0.0466	2	Cell	[212]
Gravure	2013	PET/ITO/PEDOT:PSS/P3HT:PCBM/ZnO/Al	PEDOT:PSS, P3HT:PCBM and ZnO (Gravure)	75	0.86	Module	[213]
	2014	Glass/ITO/PEDOT:PSS/PTB7:PC_61_BM/Ca/Al	PEDOT:PSS and PTB7:PC_61_BM (Gravure)	-	1.61	Cell	[151]
	2014	PET/ITO/PEDOT:PSS/P3HT:PC_61_BM/LiF/Al	PEDOT:PSS and P3HT:PC_61_BM (Gravure)	15	1.72	Cell	[231]
	2015	PET/ITO/ZnO NP/P3HT:PC_61_BM/PEDOT:PSS/Ag	ZnO NP and P3HT:PC_61_BM (Gravure). Ag (Flexography)	0.32	1.26	Cell	[89]
	2015	PET/ITO/ZnO/P3HT:PC_61_BM/PEDOT:PSS/Ag	ZnO and P3HT:PC_61_BM (Gravure). PEDOT:PSS and Ag (Rotary Screen)	96.5	1.8	Module	[138]
	2015	PET/ITO/ZnO/P3HT:PC_61_BM/PEDOT:PSS/Ag	ITO pattern, PEDOT:PSS, and Ag (Rotary screen). ZnO and P3HT:PC_61_BM (Gravure)	96.5	1.97	Module	[138]
	2016	PET/ITO/ZnO NP/P3HT:PC_61_BM/PEDOT:PSS/Ag	ZnO NP, P3HT:PC_61_BM, PEDOT:PSS and Ag (Gravure)	18	2.22	Cell	[103]
	2017	PET/ITO/ZnO/PTB7-Th:PC_71_BM/MoOx/Al	ZnO and PTB7-Th:PC_71_BM (Gravure)	0.09	6.61	Cell	[214]
	2017	PET/ITO/PEDOT:PSS/P3HT:PCBM/Li/Al	PEDOT:PSS and P3HT:PCBM (Gravure)	21.8	2	Module	[215]
	2018	Glass/ITO/ZnO/PTB7-Th:PC71BM/MoO_3_/Al	ZnO (micro-gravure)	-	6.83	Cell	[216]
	2019	PET/ITO/ZnO:PEI/P3HT:PCBM/MoO_3_/Ag	ZnO:PEI and P3HT:PCBM (Micro-gravure)	0.09	2.43	Cell	[210]
	2019	PET/ITO/ZnO:PEI/PTB7-Th/MoO_X_/Al	ZnO:PEI (Micro-gravure)	0.09	6.9	Cell	[211]
	2022	PET/ITO/ZnO/P3HT:PC_61_BM/PEDOT:PSS/Ag	ITO pattern, PEDOT:PSS and Ag (Rotary screen). ZnO and P3HT:PC_61_BM (Gravure)	15.56	2.55	Module	[232]
Inkjet	2007	Glass/TO/PEDOT:PSS/P3HT:PCBM/ZnO/Ag	Ag (Inkjet)	-	0.209	Cell	[202]
	2010	Glass/ITO/PEDOT:PSS/P3HT:PC_61_BM/LiF/Ag	PEDOT:PSS and P3HT:PC_61_BM (Inkjet)	-	3.71	Cell	[205]
	2012	PET/Ag/PEDOT:PSS/ZnO/P3HT:PCBM/PEDOT:PSS/Ag	Bottom Ag (Inkjet)	-	0.75	Cell	[208]
	2015	Glass/Ag/PEDOT:PSS/ZnO NPs/Activlink PV2000/PEDOT/Ag	PEDOT:PSS, Activlink PV2000, ZnO NP (Inkjet)	1	4.7	Cell	[201]
	2015	Glass/ITO/ZnO/P3HT:PCBM/MoO_X_/Ag	ZnO and P3HT:PCBM (inkjet)	1	2.83	Cell	[198]
	2015	Glass/Mo/Al/Mo/PEDOT:PSS/ZnO/P3HT:PCBM/MoO_X_/Ag	PEDOT:PSS, ZnO and P3HT:PCBM (inkjet)	1	2.18	Cell	[198]
	2015	Glass/ITO/ZnO/P3HT:PC_61_BM/PEDOT:PSS:MoO_3_/Ag NW	Ag (Inkjet)	-	2.71	Cell	[203]
Inkjet	2016	Glass/ITO/ZnO/P3HT:IC_60_BA/WoO_3_/PEDOT:PSS/Ag	ZnO, P3HT:IC_60_BA, WoO_3_, PEDOT:PSS and Ag (inkjet)	0.1	2.9	Cell	[143]
	2016	Glass/ITO/ZnO/P3HT:IC_60_BA/PEDOT:PSS/Ag	P3HT:IC_60_BA (Inkjet)	0.1	1.6	Cell	[143]
	2016	Glass/Ag NPs/Ag NWs/PV2000:PC_71_BM/PEDOT:PSS/Ag NW	Ag NPs, Ag NWs, PV2000:PC_71_BM, PEDOT:PSS and Ag NW (Inkjet)	1	4.3	Cell	[204]
	2018	Glass/ITO/ZnO NPs/P3HT:PC_61_BM/PEDOT:PSS/Ag	PEDOT:PSS (Inkjet)	-	1.9	Cell	[233]
	2018	PEN/Ag/ZnO/P3HT:PCBM/PEDOT:PSS	Ag, ZnO, P3HT:PCBM and PEDOT:PSS (Inkjet)	0.02	0.18	Cell	[199]
	2019	Glass/ITO/ZnO/P3HT:ICBA/MoO_3_/Ag	ZnO, P3HT:ICBA (inkjet)	0.045	4.7	Cell	[234]
	2020	Glass/ITO/ZnO/P3HT:O-IDTBR/MoOx/Ag	P3HT:O-IDTBR (Inkjet)	0.1	6.47	Cell	[200]
	2020	Glass/PEDOT:PSS/P3HT:O-iDTBR/ZnO/PEDOT:PSS	PEDOT:PSS, ZnO, P3HT:O-IDTBR, PEDOT:PSS, Ag (Inkjet)	0.1	4.73	Cell	[200]
	2021	glass/ITO/PEDOT:PSS/p-DTS(FBTTh2)2:PC_71_BM/Ca/Al	p-DTS(FBTTh2)2:PC_71_BM (inkjet)	-	7.3	Cell	[207]
	2022	Glass/ITO/PEDOT:PSS:Graphene/PTB7:PCBM/LiF/Al	PEDOT:PSS:Graphene and PTB7:PCBM (Inkjet)	0.7	1.12	Cell	[206]
Pad	2008	Glass/ITO/ZnO/P3MHOCT + Zinc/PEDOT:PSS/Ag	P3MHOCT + Zinc (Pad)	-	0.07	Cell	[209]
Rod	2011	Glass/AgNWs-TIO_2_/PEDOT:PSS/P3HT:PC_61_BM/Ca/Al	AgNWs (Rod)	-	3.4	Cell	[135]
	2017	Glass/ITO/PEDOT:PSS/P3HT:PCBM/LiF/Al	PEDOT:PSS and P3HT:PCBM (Rod)	0.1	1.9	Cell	[235]
Screen	2004	PET/ITO/Ag/MEH-PPV/Al	Ag and MEH-PPV (Screen)	0.12	0.046	Cell	[222]
	2009	Glass/ITO/PEDOT:PSS/P3HT:PCBM/Al	P3HT:PCBM (Screen)	3	2.3845	Cell	[225]
	2018	Steel/Insulator/Al/ZnO/P3HT:PC61BM/PEDOT:PSS/Al	PEDOT:PSS (Screen)	0.09	0.67	Cell	[223]
Slot-die	2009	PET/ITO/ZnO/P3HT/PEDOT:PSS/Ag	ZnO, P3HT, PEDOT:PSS and Ag (Slot-die)	120	0.84	Module	[236]
	2010	PET/ITO/ZnO NP/P3HT:PCBM/PEDOT:PSS/Ni	ZnO NP, P3HT:PCBM and PEDOT:PSS (Slot-die). n-octanol (Flexographic). Nickel (Rotary screen)	15	2.75	Cell	[114]
	2010	PET/ITO/ZnO/P3HT:PC_61_BM/PEDOT:PSS/Ag	ZnO, P3HT:PC_61_BM and PEDOT:PSS (Slot-die). Ag (Rotary screen)	360	1.18	Module	[90]
	2010	PET/ITO/ZnO/P3HT:PC_61_BM/PEDOT:PSS/Ag	ZnO, P3HT:PC_61_BM and PEDOT:PSS (Slot-die). Ag (Rotary screen)	160	1.22	Module	[90]
	2010	PET/ITO/ZnO/P3HT:PC_61_BM/PEDOT:PSS/Ag	ZnO, P3HT:PC_61_BM and PEDOT:PSS (Slot-die). Ag (Rotary screen)	96	1.79	Module	[90]
Slot-die	2011	PET/Cr/Al/Cr/P3HT:PCBM/PEDOT:PSS/Au	P3HT:PC61BM and PEDOT:PSS (Slot-die)	20.6	1.40	Module	[165]
	2012	PET/Ag/PEDOT:PSS/ZnO NPs/P3HT:PC_61_BM/PEDOT:PSS/Ag	PEDOT:PSS, P3HT:PC_61_BM and ZnO NP (Slot-die)	1	1.5	Cell	[167]
	2013	ITO/ZnO/PSBTBT:PDI-DTT/PEDOT:PSS/Ag	PSBTBT:PDI-DTT (Slot-die)	4.2	0.204	Cell	[171]
	2013	Ag grid/PEDOT:PSS/ZnO/P3HT:PCBM/PEDOT:PSS/Ag grid	Bottom Ag (Flexography), PEDOT:PSS (Rotary Screen), ZnO and Active (Slot-die), Top Ag (Flatbed screen)	24	2.09	Cell	[180]
	2013	Ag grid/PEDOT:PSS/ZnO/P3HT:PCBM/PEDOT:PSS/Ag grid	Bottom Ag (Flexography), PEDOT:PSS (Rotary Screen), ZnO and Active (Slot-die), Top Ag (Rotary screen)	24	1.84	Cell	[180]
	2013	Ag grid/PEDOT:PSS/ZnO/P3HT:PCBM/PEDOT:PSS/Ag grid	Bottom Ag (Flexography), PEDOT:PSS (Rotary Screen), ZnO and Active (Slot-die), Top Ag (Inkjet)	24	0.54	Cell	[180]
	2013	Ag grid/PEDOT:PSS/ZnO/P3HT:PCBM/PEDOT:PSS/Ag grid	Bottom Ag (Flexography), PEDOT:PSS (Rotary Screen), ZnO and Active (Slot-die), PEDOT:PSS (Rotary Scree), Top Ag (Flexography)	24	0.12	Cell	[180]
	2014	PET/ITO/Ag/ITO/AZO/P3HT:PCBM/PEDOT:PSS/Ag	AZO and PEDOT:PSS (Blade), P3HT:PCBM (Slot-die)	2.5	3.03	Cell	[174]
	2015	PET/AgNW/ZnO/PBDPTTTz-4:PC61BM/PEDOT:PSS/Ag	AgNW, PEDOT:PSS and PBDPTTTz-4:PC61BM (Slot-die). Ag (Flexography)	1	3.3	Cell	[168]
	**2017**	**PET/ITO/AZO:PEIE/P3HT:PC_71_BM/MoO_3_/Ag**	**AZO and P3HT:PC_71_BM (Slot-die)**	**2**	**5.7**	**Cell**	[86]
	2017	PET/ITO/AZO/PTB7:PC_71_BM/MoOx/Ag	AZO and PTB7:PC_71_BM (Slot-die)	0.3	7.32	Cell	[86]
	2018	PET/Ag grid/PEDOT:PSS/ZnO/PI-4 ink/HTL/Ag Vacuum	PI-4 ink and HTL (Slot-die)	-	0.669	Cell	[166]
	2018	PET/ZnO NPs/PPDT2FBT:PC_71_BM/MoOx/Al	ZnO NPs and PPDT2FBT:PC_71_BM (Slot-die)	0.1	7.1	Cell	[63]
	2019	glass+C14:G14s/ITO/ZnO/PEIE/PPDT2FBT:PC_61_BM/MoOX/Ag	PPDT2FBT:PC_61_BM (Slot-die)	0.09	8.48	Cell	[67]
	2019	glass/ITO/ZnO/PEIE/PPDT2FBT:ITIC-F/MoOX/Ag	PPDT2FBT:ITIC-F (Slot-die)	0.09	8.48	Cell	[67]
	2019	PET/Ag/ZnO/PBDB-T:ITIC/HTL solar/CPP PEDOT:PSS	ZnO, PBDB-T:ITIC, HTL and PEDOT:PSS (Slot-die)	0.054	5.07	Cell	[113]
	2019	PET/Ag/PEDOT:PSS/P3HT:ICxA/ZnO/Al	Ag (Flexography), PEDOT:PSS, P3HT:ICxA and ZnO (Slot-die)	4	0.33	Cell	[173]
Slot-die	2019	PET/ITO/ZnO/PTB7-Th+p-DTS(FBTTH2)2:PC_71_BM/MoOx/Ag	ZnO, PTB7-Th+p-DTS(FBTTH2)2:PC_71_BM (Slot-die)	0.75	7.32 ± 0.431	Cell	[163]
	**2019**	**PET/ITO/ZnO/PTB7-Th+p-DTS(FBTTH2)2:PC_71_BM/MoOx/Ag**	**ZnO, PTB7-Th+p-DTS(FBTTH2)2:PC_71_BM (Slot-die)**	**1**	**7.11 ± 0.263**	**Cell**	[163]
	**2019**	**PET/ITO/ZnO/PTB7-Th+p-DTS(FBTTH2)2:PC_71_BM/MoO_X_/Ag**	**ZnO, PTB7-Th+p-DTS(FBTTH2)2:PC_71_BM (Slot-die_**	**2**	**6.79 ± 0.212**	**Cell**	[163]
	2019	Glass/ITO/ZnO/PBDB-T:iIEICO-4F/MoO_3_/Al	PBDB-T:iIEICO-4F (Slot-die)	-	12.2 ± 0.17	Cell	[65]
	2019	Glass/ITO/ZnO/PTB7-Th:PC_71_BM:COi8DFIC/MoO_3_/Al	PTB7-Th:PC_71_BM:COi8DFIC (Slot-die)	0.1	13.5	Cell	[87]
	**2019**	**Glass/ITO/ZnO/PTB7-Th:PC_71_BM:COi8DFIC/MoO_3_/Al**	**PTB7-Th:PC_71_BM:COi8DFIC and ZnO (Slot-die)**	**30**	**8.6**	**Module**	[87]
	2019	PET/TCO/ZnO/PTB7-Th:PC_71_BM:COi8DFIC/MoO_3_/Al	PTB7-Th:PC_71_BM:COi8DFIC and ZnO (Slot-die)	0.14	9.57	Cell	[87]
	2020	flextrode/P3HT:O-IDTBR/PEDOT:PSS/Ag	P3HT:O-IDTBR and PEDOT:PSS (Slot-die). Ag (Flexography)	1	3.26 ± 0.14	Cell	[169]
	2020	PET/ZnO/PV2001:PC_61_BM/PEDOT:PSS/Ag	ITO patterning (Rotary screen). ZnO and PV2001:PC_61_BM (Slot-die). PEDOT:PSS and Ag (Flatbed screen)	-	4.6	Module	[64]
	**2021**	**PET/ITO/Ag/ITO/PEI/RaynergyTek:PC_61_BM/PEDOT:PSS/Ag**	**PEI, RaynergyTek:PC_61_BM and PEDOT:PSS (Slot-die)**	**7.2**	**5.6 ± 0.6 (6.0)**	**Module**	[139]
	2021	PET/TCE/ZnO NPs/PM6:Y6:IT-4F/MoO_3_/Ag	ZnO NPs and PM6:Y6:IT-4F (Slot-die)	0.14	10.2	Cell	[170]
	2021	Glass/ITO/ZnO/PTB7-Th:IEICO-4F/PEDOT:PSS and MoO_3_/Ag	PEDOT:PSS, ZnO, PTB7-Th:IEICO-4F (Slot-die)	0.1	11	Cell	[162]
	**2021**	**PET/ITO/Ag/ITO/PEI/M3:PC60BM/PEDOT:PSS/Ag**	**PEI, M3:PC60BM and PEDOT:PSS (Slot-die)**	**7.2**	**5.6 ± 0.6**	**Module**	[139]
	**2021**	**Glass/ITO/PEDOT:PSS/PTB7-Th:IEICO-4F/AZO/Ag**	**PEDOT:PSS, PTB7-Th:IEICO-4F and AZO (Slot-die)**	**1**	**9.6**	**Cell**	[162]
	2021	Glass/ITO/PEDOT:PSS/PTB7-Th:IEICO-4F/AZO/Ag NWs	PEDOT:PSS, PTB7-Th:IEICO-4F and AZO (Slot-die), Ag NWs (blade)	0.2	8.8	Cell	[162]
	2021	PET/ITO/ZnO/PV2000:PC_71_BM/PEDOT:PSS/Ag	ZnO, PV2000:PC_71_BM and PEDOT:PSS (Slot-die)	48	2.2	Module	[94]
	2022	Glass/ITO/PEDOT:PSS/PM6:Y6C12/PFNBr/Ag	PEDOT:PSS, PM6:Y6C12 and PFNBr (Slot-die)	0.14	10.1 ± 0.6	Cell	[164]
	2022	Glass/ITO/SnO2/PDIN-H/PTQ10:Y6-C12/MoO_X_/Ag	SnO2, PDIN-H, PTQ10:Y6-C12 and PEDOT:PSS (Slot-die)	0.12	11.26 ± 0.49	Cell	[59]
Slot-die	2022	Glass/ITO/SnO2/PDIN-H/PTQ10:Y6-C12/PEDOT:PSS/Ag	SnO2, PDIN-H, PTQ10:Y6-C12 and PEDOT:PSS (Slot-die)	0.12	9.38 ± 0.2	Cell	[59]
	2022	Glass/ITO/PEDOT:PSS/PM6:Y6C12/PDIN-EH/Ag	PEDOT:PSS, PM6:Y6C12 and PDIN-EH (Slot-die)	0.12	11.9 ± 0.2	Cell	[104]
	**2022**	**Glass/ITO/PEDOT:PSS/PM6:Y6C12/PDIN-EH/Ag**	**PEDOT:PSS, PM6:Y6C12 and PDIN-EH (Slot-die)**	**2**	**7.5**	**Module**	[104]
	2022	PET/ITO/PEDOT:PSS/PM6:Y6C12/PDIN-EH/Ag	PEDOT:PSS, PM6:Y6C12 and PDIN-EH (Slot-die)	16	2.4	Module	[104]
	2022	PET/ITO/ZnO NPs/PPDT2FBT:PC61BM/MoOx/Al	ZnO NPs, PPDT2FBT:PC61BM (Slot-die)	0.1	8.49 ± 0.07	Cell	[88]
	**2022**	**PET/Ag NW/SnO_2_ NPs/PV2000:PC_61_BM/PEDOT:PSS/Ag**	**Ag NWs, SnO_2_ NPs, PV2000:PC_61_BM, PEDOT:PSS, Ag (Slot-die)**	**9.3**	**5.10**	**Module**	[237]
	**2022**	**Glass/ITO/PEDOT:PSS/PM6:T8/PNDIT-F3N/Ag**	**PM6:T8 (slot-die)**	**7.5**	**14.6**	**Module**	[42]
	2022	Glass/ITO/PEDOT: PSS/PM6:Y6/PFN-Br/Ag	PM6:Y6 (Slot-die)	-	16.22	Cell	[181]
	2023	PET/ITO/ETL/PTB7:PC_71_BM/MoO_3_/Ag	AZO:PEIE and PTB7:PC_71_BM (Slot-die)	0.3	6.07 ± 0.19	Cell	[175]
	**2023**	**PET/ITO/ETL/PTB7:PC_71_BM/MoO_3_/Ag**	**AZO:PEIE and PTB7:PC_71_BM (Slot-die)**	**4**	**5.91 ± 0.17**	**Cell**	[175]
	**2023**	**PET/ITO/ETL/PTB7:PC_71_BM/MoO_3_/Ag**	**AZO:PEIE and PTB7:PC_71_BM (Slot-die)**	**2**	**5.80 ± 0.18**	**Module**	[175]
	2023	PET/IMI/SnO_2_/P3HT:o-IDTBR/PEDOT:PSS/AgNW	SnO_2_, P3HT:o-IDTBR, PEDOT:PSS and AgNWs (Slot-die)	0.1375	3.50	Cell	[176]
	**2023**	**PET/ITO/ZnO/PBDB-T:ITIC/MoO_X_/Ag**	**ZnO and PBDB-T:ITIC (Slot-die)**	**4**	**5.70**	**Cell**	[178]
	**2023**	**PET/TCO/ZnO NPs/PV-X ink/HTL3 ink/Ag**	**ZnO NPs, PV-X ink and HTL3 ink (Slot-die)**	**45**	**6.20**	**Module**	[238]
	**2023**	**PET/Ag/PEDOT:PSS/ZnO/PM6:L8-BO/MoOx/Ag**	**PEDOT:PSS, ZnO and PM6:L8-BO (Slot-die)**	**1.036**	**12.17**	**Cell**	[239]
	2024	Glass/ITO/SnO_2_/PBI-Y/PM6:Y6C12/MoO_X_/Ag	SnO_2_, PBI-Y and PM6:Y6C12 (Slot-die)	0.14	10.60	Cell	[179]
Spray	2013	Glass/ITO/PEDOT:PSS/CuPc:C_60_/Al	CuPc/C_60_ (Spray)	-	1.08	Cell	[195]
	2013	Glass/ITO/PEDOT:PSS/P2:PC_71_BM/Ca/Al	P2:PC_71_BM (Spray)	0.045	5	Cell	[183]
	2013	Glass/ITO/PEDOT:PSS/P3HT:PCBM/Bphen/Ag	P3HT:PC61BM (Spray)	-	2.91	Cell	[185]
	2013	Glass/ITO/PEDOT:PSS/P3HT:PCBM/Ca/Ag	P3HT:PCBM (Spray)	-	3.9 ± 0.2	Cell	[187]
Spray	2013	Glass/ITO/PEDOT:PSS/P3HT:PC_61_BM/LiF/Al	P3HT:PC_61_BM (Spray)	0.25	2.59	Cell	[197]
	2015	Glass/ITO/PEDOT:PSS/P3HT:PC_61_BM/Ca/Al	P3HT:PC_61_BM (Spray)	0.1	3.33 ± 0.16	Cell	[194]
	2017	Glass/ITO/PEDOT:PSS/PTB7:PC_71_BM/LiF/Al	PTB7:PC_71_BM (Spray)	-	5.6	Cell	[83]
	2018	Glass/ITO/ZnO/P3HT:PC61BM/PEDOT:PSS	ZnO, P3HT:PCBM and PEDOT:PSS (Spray)	4	2.44	Cell	[188]
	2018	Textile/Ag/ZnO/P3HT:ICBA/PEDOT:PSS/AgNW	ZnO, P3HT:ICBA, PEDOT:PSS, AgNW (Spray)	0.03	1.23	Cell	[191]
	2018	Glass/ITO/PEDOT:PSS/P3HT:PC_71_BM/Al	P3HT:PC_71_BM (Spray)	-	2.17	Cell	[193]
	2018	Glass/ITO/PEDOT:PSS/PffBT4T2OD:PC_71_BM/PEIE/V_2_O_5_/Ag	PffBT4T2OD:PC_71_BM, PEIE and V_2_O_5_ (Spray)	-	3.56 ± 0.25	Cell	[93]
	2018	Glass/Graphene/PEDOT:PSS/PffBT4T2OD:PC_71_BM/PEIE/V_2_O_5_/Ag	PffBT4T2OD:PC71BM, PEIE and V_2_O_5_ (Spray)	-	2.82 ± 0.15	Cell	[93]
	2019	PEN/Graphene/MoOx/PEDOT:PSS/P3HT:PC_61_BM/Ca/Ag	P3HT:PC_61_BM (Spray)	2	1.5	Cell	[189]
	2019	Glass/ITO/PEDOT:PSS/PBDTTT-EFT:PC_71_BM/Ca/Al	PEDOT:PSS and PBDTTT-EFT:PC_71_BM (Spray)	-	7.5	Cell	[192]
	2020	Glass/ITO/PEDOT:PSS/P3HT:PC_71_BM/LiF/Al	P3HT:PC_71_BM (Spray)	-	3.48	Cell	[182]
	2020	Glass/ITO/PEDOT:PSS/P3HT:PC_61_BM/LiF/Al	P3HT:PC_71_BM (Spray)	-	1.97	Cell	[190]
	2020	Glass/ITO/PEDOT:PSS/PTB7-Th:FOIC/PDINO/Al	PTB7-Th:FOIC	0.048	9.45	Cell	[240]
	2020	Glass/ITO/PEDOT:PSS/PTB7-Th:FOIC/PDINO/Al	PEDOT:PSS. PTB7-Th:FOIC, PDINN (Spray)	0.048	8.71	Cell	[240]
	2020	Glass/ITO/PEDOT:PSS/P3HT:PC_61_BM/LiF/Al	P3HT:PC_61_BM (Spray)	0.04	1.97	Cell	[190]
	2020	Glass/ITO/PEDOT:PSS/P3HT:PC_71_BM/LiF/Al	P3HT:PC_71_BM (Spray)	0.04	3.48	Cell	[182]
	2021	Glass/ITO/PEDOT:PSS/PM6:N3/PDINN/Ag	PM6:N3 (Spray)	0.048	15.98	Cell	[196]
	2021	Glass/ITO/PEDOT:PSS/PM6:N3/PDINN/Ag	PEDOT:PSS, PM6:N3, PDINN (Spray)	0.048	14.55	Cell	[196]
	2022	Glass/ITO/PEDOT:PSS/PTB7-Th:PC_71_BM/Ca/Al	PTB7-Th:PC_71_BM (Spray)	0.0575	7.36	Cell	[241]
	2024	Glass/ITO/ZnO/P3HT:PCBM/MoO_X_/Ag	ZnO, P3HT:PCBM, MoO_X_ (Spray)	0.3	2.97	Cell	[242]

**Table 2:** Technical comparison of different printing/coating techniques. The table was adapted from Krebs 2009 [119].

Conditions	R2R Compatible	Pattern	Ink Preparation	Wastage	Web Speed	Wet Thickness (um)	Ink Viscosity
Spin	N	0	1	5	-	0–100	1
Blade	Y	0	2	2	2–4	0–100	1
Slot-die	Y	1	2	1	3–5	10–250	2–5
Spray	Y	0	2	3	1–4	1–500	2–3
Inkjet	Y	2	3	1	1	1–500	1
Pad	Y	2	5	1	1–2	5–250	1
Gravure	Y	2	4	1	1–3	5–80	1–3
Flexography	Y	2	4	1	1–3	5–200	1–3
Screen	Y	2	4	1	1–4	10–500	3–5

Pattern: 0 (zero-dimensional), 1 (one-dimensional), and 2 (two-dimensional). Ink preparation: 1 (simple), 2 (moderate), 3 (demanding), 4 (difficult), and 5 (critical). Ink waste: 1 (none), 2 (little), 3 (some), 4 (considerable), and 5 (significant). Web Speed: 1 (very slow), 2 (slow < 1 m min^−1^), 3 (medium 1–10 m min^−1^), 4 (fast 10–100 m min^−1^), and 5 (very fast 100–1000 m min^−1^). Ink viscosity: 1 (very low < 10 cP) 2 (low 10–100 cP), 3 (medium 100–1000 cP), 4 (high 1000–10,000 cP), and 5 (very high 10,000–100,000 cP).

**Table 3:** Highlighting advantages and disadvantages of printing and coating techniques for OPV fabrication.

Technique	Advantages	Disadvantages	Use
Blade coating	Demonstrated to fabricate high-performance/large-area rigid OPV devices. Relatively simple coating method. Ideal for sheet-to-sheet processing.	Not well suited for roll-to-roll OPV fabrication and requires post-processing for patterning	Large-area coating (interface and active material) over rigid substrates using sheet-to-sheet processing
Flexography printing	Excellent at printing of low–medium flexible devices at medium printing speeds, achieving relatively thick films.	Difficulty in controlling morphology and film quality without engineering solutions.	Electrode printing for flexible substrates via roll-to-roll processing.
Gravure printing	Excellent at printing of low–medium flexible devices at medium printing speeds.	Difficulty in controlling morphology and film quality without engineering solutions. Limited in thickness.	Interfacial printing for flexible substrates via roll-to-roll processing.
Inkjet printing	Excellent for laboratory scales that require high-precision patterning. Patterns can be easily changed compared to other printing methods.	Too-slow coating speed to be viable for large-scale OPV fabrication, as well as limited by low viscosity.	Laboratory-scale devices that require high precision of patterning (interfacial and active materials).
Pad printing	With technique optimisation, the technique could allow for repeatable pattern printing.	Relatively complex setup for small area coating. Difficulty with reproducibility.	Not ideal for OPV fabrication.
Rod coating	Relatively simple method for coating low viscous increases.	Limited to laboratory and small-scale coatings. Difficult to coat with high viscous inks.	Small-scale coating of low viscous (interfacial ad active materials) inks.
Screen printing	Allows for the printing of high viscous inks at large layer thicknesses. Can be designed for sheet-to-sheet or roll-to-roll processing.	Not ideal for low-viscosity inks, with the risk of uncontrol film deposition as the ink passed through the screen.	Large-area printing of electrodes for both sheet-to-sheet and roll-to-roll processes.
Slot-die coating	Demonstrated for fabrication of high-performing flexible and rigid solar cells at both laboratory- and small-scale conditions.	High-performance devices/modules not yet demonstrated on large scale/beyond small scale.	Large-area coating (interfacial and active materials) over a flexible substrate using roll-to-roll processing.
Spray coating	Useful for coating thin films over uneven surfaces, with the ability to coat interfacial and active layer materials.	Limited to ink concentration and viscosity, leading to thick films require extended coating time.	Small to medium area coating (interfacial and active materials) for non-uniform surfaces.

## Data Availability

Not applicable.
